# Psychological interventions for weight reduction and sustained weight reduction in adults with overweight and obesity: a scoping review

**DOI:** 10.1136/bmjopen-2023-082973

**Published:** 2024-12-02

**Authors:** Oliver Hamer, Jennifer A Kuroski, Emma P Bray, Cath Harris, Amy Blundell, Emma Schneider, Caroline Watkins

**Affiliations:** 1 University of Central Lancashire, Preston, UK; 2 Blackburn with Darwen Borough Council, Blackburn, UK; 3 Liverpool University Hospitals NHS Foundation Trust, Liverpool, UK

**Keywords:** Obesity, Body Mass Index, Psychological Stress, Health Services, PUBLIC HEALTH, THERAPEUTICS

## Abstract

**Abstract:**

**Introduction:**

Overweight and obesity are growing public health problems worldwide. Both diet and physical activity have been the primary interventions for weight reduction over the past decade. With increasing rates of overweight and obesity, it is evident that a primary focus on diet and exercise has not resulted in sustained obesity reduction within the global population. There is now a case to explore other weight management strategies such as psychological therapies. However, there is a dearth of literature that has mapped the types of psychological interventions and the characteristics of these interventions as a means of achieving weight reduction.

**Objectives:**

The key objectives focused on mapping the types and characteristics of psychological interventions versus usual care for weight reduction and sustained weight reduction in adults with overweight or obesity. The study followed the scoping review methodology by Arksey and O’Malley and was reported in accordance with the Preferred Reporting Items for Systematic Reviews and Meta-Analyses extension for Scoping Reviews guidelines.

**Eligibility criteria:**

Intervention studies were included if participants were 18 years and over, classified as overweight or obese (body mass index ≥25 kg/m^2^) and had received a psychological therapy intervention. Studies were excluded if they included a comparison with other active lifestyle interventions (unless classified as usual care), were not available in English, were not full-text articles or were non-peer-reviewed articles.

**Sources of evidence:**

Six electronic databases were searched from inception to April 2023 to identify relevant articles.

**Charting methods:**

The study employed a systematic charting method and narrative synthesis to organise and synthesise the data.

**Results:**

A total of 31 studies met the eligibility criteria and were included in the review. 13 unique psychological interventions for weight reduction in adults with overweight or obesity were identified, with cognitive–behavioural therapy and motivational interviewing being the most common. Eight types of usual care were identified, which largely included education and training on nutrition and physical activity. Gaps in the current research were also identified.

**Conclusion:**

The findings highlighted several gaps within the existing literature, largely due to a lack of evidence relating to adults with low socioeconomic status, non-white participants, individuals under 40 years of age and the integration of digital health technologies.

**STRENGTHS AND LIMITATIONS OF THIS STUDY**

- The scoping review used a robust search strategy including six electronic databases and examined the reference lists of related studies to identify other relevant studies.
- The scoping review has been codeveloped with a public advisor who has over 10 years experience living with obesity.
- The scoping review provides a comprehensive overview of the types of psychological interventions for weight reduction and sustained weight reduction in adults with overweight or obesity.
- The scoping review was limited to comparisons with other psychological interventions, usual care or no intervention and did not include a comparison with other active lifestyle interventions unless classified as usual care.

## Introduction

 Obesity is a growing public health problem worldwide.^1^ Globally, the prevalence of obesity among the adult population (ie, individuals aged 18 and above) has risen to approximately 650 million.^2^ A further 1.25 billion adults are estimated to be overweight, making up nearly 40% of the global population.^3^ This is an increasing concern given that excessive body weight is known to increase the risks of a wide variety of comorbidities (eg, cardiovascular disease, type 2 diabetes, cancer, hypertension and musculoskeletal disorders).^4 5^ Overweight and obesity are defined by a body mass index (BMI) of 25–29.9 kg/m^2^ and 30 kg/m^2^ or greater, respectively.^2^ Both overweight and obesity have multifaceted aetiologies, with individual (eg, genetics, medical conditions, stress and lifestyle factors) and environmental factors (eg, socioeconomic aspects and urbanisation) contributing to prevalence.^1 6^ Over the past two decades, although most interventions for weight reduction have focused on individual-level lifestyle modifications (eg, increasing physical activity), some have included surgery (bariatric surgery) and pharmacological medication.^6^,^8^ These have been accompanied by policy-based approaches (eg, sugar taxes, regulation of advertising, reform of licensing of food outlets)^7 8^ aiming to address obesity at the population level.

Both energy input from food consumption and energy expenditure from physical activity (ie, energy balance) are key factors to weight maintenance and, therefore, diet (energy intake) and physical activity (energy output) are key behavioural determinants of obesity.^9^ Over the last 20 years, diet and physical activity have been the primary interventions recommended for weight loss.^10^ However, as many individuals regain the weight lost and with global overweight and obesity rates continuing to rise, it is evident that this approach has not been wholly effective.^11 12^ While increasing energy expenditure and reducing dietary intake^13^ are critical for weight loss (in most cases), they often ignore the complex psychological, emotional, environmental and behavioural factors that underpin overweight and obesity.^214^,^16^ These factors can contribute to disordered eating, low self-esteem, depression, anxiety and stress, which in turn can lead to further weight gain (eg, environmental factors such as access to safe and non-discriminatory recreational spaces and access to healthy, affordable food may exacerbate psychological struggles).^14 17^

In recent years, there has been increasing recognition of the importance of psychological interventions for weight reduction in adults with overweight and obesity.^18^ These interventions support individuals to address the underlying psychological factors (eg, low mood, low self-efficacy, anxiety, body image concerns, emotional eating) that contribute to overweight and obesity, by refining skills and strategies to cope with negative emotions, modifying unhelpful behaviours and encouraging healthy lifestyle changes.^18 19^ They also aim to increase self-efficacy, self-regulation and self-awareness, which are critical for long-term weight reduction.^20^ By targeting these psychological factors, interventions may be more effective for long-term weight reduction compared with diet and physical activity interventions alone.^21^ These interventions include a variety of components (eg, cognitive behaviour change, mindfulness, relaxation and motivation), are conducted individually or in groups and are typically delivered in clinical, community, workplace or online settings.^19 20^

Within the last two decades, research in the field of weight reduction (ie, the intentional efforts to decrease body weight) and weight management (ie, long-term approach to maintaining a healthy weight) has rapidly evolved with a particular focus on psychological interventions.^22^ This is likely due to the recognised need for personalised approaches to weight reduction in which psychological interventions have shown to be effective.^22 23^

As a consequence of the greater interest in this area of research, numerous systematic reviews have been conducted to assess the effectiveness of psychological interventions for weight reduction in adults with overweight and obesity.^19 20^ However, these reviews are limited by population (including only adults with specific medical conditions related to eating disorders),^23^ type of interventions (including only one type of psychological therapy intervention of cognitive–behavioural therapy (CBT))^19^ or being substantially outdated.^20^ With the rapid increase of emerging studies (including novel therapeutic techniques) and growing rates of overweight and obesity, a scoping review is now needed to map the different types of psychological interventions and their core characteristics, while identifying evidence gaps for future research in this field.^24^

### Aim

To explore psychological interventions for adults with overweight or obesity as a means of achieving weight reduction and sustained weight reduction.

### Objectives

1. To generate a comprehensive map of the types of psychological interventions for weight reduction and sustained weight reduction in adults with overweight or obesity.
2. To identify the core intervention characteristics including theoretical foundations, techniques, setting, duration, mode, the number of sessions and provider of psychological interventions for weight reduction and sustained weight reduction in adults with overweight or obesity.
3. To identify study characteristics such as length of follow-up, and outcomes which include psychological interventions for weight reduction and sustained weight reduction in adults with overweight or obesity.
4. To generate a map of the types of usual care for weight reduction and sustained weight reduction in adults with overweight or obesity.
5. To identify the core characteristics including components, setting, duration, mode and providers of usual care for weight reduction and sustained weight reduction in adults with overweight or obesity.
6. To identify evidence gaps and areas for future research in the field of psychological interventions for weight reduction and sustained weight reduction in adults with overweight and obesity.

## Methods

### Study design

The study followed a scoping review design, adhering to the framework set out by Arksey and O’Malley, which recommends a five-stage review process^25^:

- Stage 1: identifying the research question.
- Stage 2: identifying relevant studies.
- Stage 3: study selection.
- Stage 4: charting the data.
- Stage 5: collating, summarising and reporting the results.

We did not implement the optional sixth stage set out by Arksey and O’Malley as the first five stages were adequate to satisfy the objectives of this review.^26^ The study was reported in accordance with the Preferred Reporting Items for Systematic Reviews and Meta-Analyses extension for Scoping Reviews (PRISMA) guidelines (see online supplemental material1).^27 28^ In addition, the scoping review followed a protocol that was developed and published a priori (DOI: 10.1136/bmjopen-2023-075364).

### Search strategy

Six databases were searched to identify relevant articles:

1. Cochrane Central Register of Controlled Trials (CENTRAL via Wiley).
2. WHO International Clinical Trials Registry Platform (ICTRP).
3. EMBASE (Ovid).
4. MEDLINE (Ovid).
5. CINAHL (EBSCOhost).
6. PsycINFO (EBSCOhost).

We searched the above databases and trial registries from their inception to April 2023 (searches conducted on 19 April 2023). A search of clinical trials was conducted through the CENTRAL and ICTRP databases to identify ongoing clinical trials. In addition, we checked the reference lists of all included studies and relevant review articles for additional studies.

The search strategy was conducted in each database based on the following:

- Population: Adults with overweight or obesity (aged ≥18, with BMI ≥25 kg/m^2^).
- Intervention: Psychological interventions (eg, CBT, mindfulness, motivational interviewing (MI)).
- Study type: Randomised controlled trials (RCTs) or non-randomised comparative intervention studies.

The search strategy was developed by the research team in collaboration with an expert information specialist, adapted for each database (see online supplemental material 2) for Medline, Ovid example). The search strategy was based on the strategy outlined in the most relevant Cochrane review by Shaw *et al*^20^, but with the addition of further search terms (identified by the research team), and the Canada’s drug and health technology research working group (Canadian Agency for Drugs and Technologies in Health (CADTH)) search filter to identify randomised and non-RCTs.^20 29^

### Study selection

**Figure 1:**
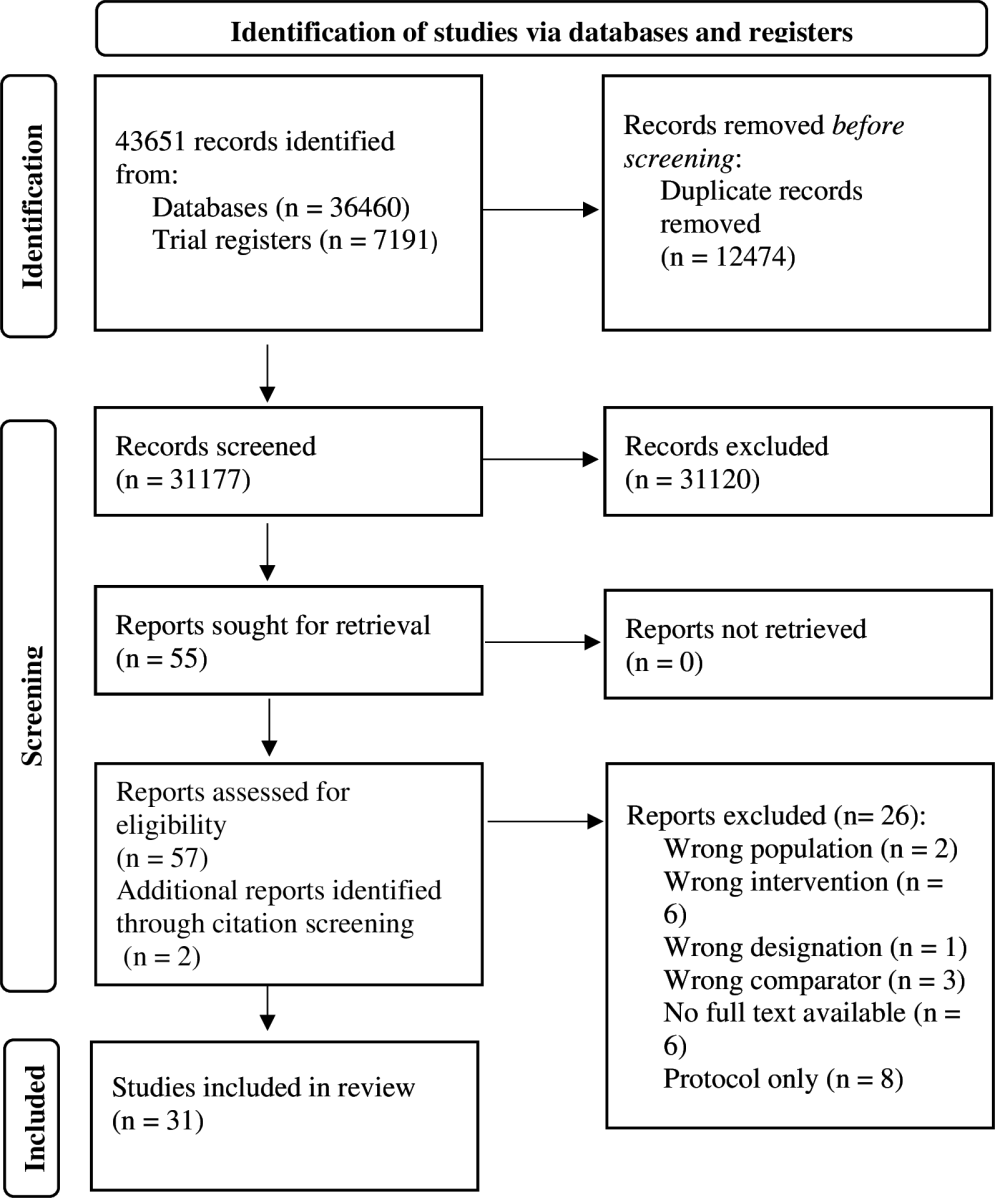
PRISMA flow diagram. PRISMA, Preferred Reporting Items for Systematic Reviews and Meta-Analyses.

All articles identified by the electronic database searches were imported into Rayyan (web application). Once imported, the duplicate removal function within Rayyan was used to remove duplicate articles. Following the removal of duplicates, one reviewer independently screened the titles and abstracts of the search results for eligible studies. The reviewer used the Rayyan software to categorise studies into labelled folders: includes, excludes or undecided. A second reviewer independently screened studies which were initially categorised as undecided. Once screening of titles and abstracts was completed (establishing includes and excludes), full texts of articles deemed to be eligible after initial screening were retrieved and uploaded onto Rayyan. Following this, two reviewers independently screened the full texts of all potentially eligible studies within the Rayyan software (using the blinding function). Once full-text screening was complete, the two reviewers convened to determine agreement on the eligibility of the articles. Disagreement regarding one article was resolved through discussion with a third reviewer. Following agreement on eligible full texts, both reviewers independently conducted citation screening of the articles to identify any further studies meeting the inclusion criteria. All full-text articles that met the inclusion criteria were charted to summarise the findings. The study selection process was recorded in sufficient detail using a PRISMA flow diagram, reporting reasons for exclusion (see figure 1 and online supplemental material 3).

### Data extraction

One reviewer independently extracted data for the included studies using a piloted data extraction form. A second reviewer independently checked and verified all the data extracted by the first reviewer. Data were extracted on the following: study methods, participants, interventions (eg, type, setting and components), outcomes and any other information judged to be important to meet the objectives of the study.

### Inclusion criteria

While a scoping review traditionally encompasses a wide range of literature, specific criteria were employed to identify relevant articles for the aim and objectives of the study.

#### Types of studies

RCTs and non-randomised comparative intervention studies were included. We only included studies reported in full text. If an abstract (eg, conference abstract) meeting the inclusion criteria was identified, the study authors were contacted to establish whether a full text was available.

#### Types of participants

Studies were included if the participants were ≥18 years and were classified as overweight or obese (BMI ≥25 kg/m^2^, height/weight could be measured, self-reported or self-measured). Studies were also included when a sample included participants with other classifications of BMI (eg, underweight or healthy weight, BMI <25 kg/m^2^) if data relating to those with overweight or obesity could be extracted separately. Studies were also included when participants with overweight or obesity had other comorbidities (eg, musculoskeletal pain, osteoarthritis, diabetes, hypertension, arthritis, hyperuricaemia, gall bladder disease), as BMI greater than 25 kg/m^2^ has been associated with the incidence of multiple comorbidities.^30^

#### Types of interventions

Psychological therapy interventions were defined as those interventions that involved meeting with a therapist (healthcare professional competent in providing psychological therapy) to discuss feelings and thoughts, and how these affect behaviour and well-being.^31^ All psychological interventions were considered for inclusion. Some of these interventions included CBT, mindfulness, psychodynamic therapies, MI, prolonged exposure therapy, hypnotherapy, psychotherapy, rationale emotional therapy and psychodynamic therapy. Interventions delivered one-to-one or in group setting were included.

We only included studies whereby the intervention was a single-component psychological therapy intervention, excluding those with multiple intervention components (eg, psychological therapy with integrated physical activity). In addition, we only included studies of psychological therapies that were delivered by a healthcare professional and were designed to reduce weight or increase health promoting behaviour for the purpose of weight reduction and sustained weight reduction (ie, eating behaviour or physical activity). Weight reduction was defined as an intentional effort to decrease body weight, while sustained weight reduction was defined as the successful long-term maintenance of a lower body weight following initial weight loss.

To identify psychological interventions for weight reduction and sustained weight reduction, we included studies with the following comparisons:

- Psychological therapy versus no intervention (ie, no intervention of any type).
- Psychological therapy versus usual care, standard care or treatment as usual (as defined by study authors).
- Comparisons between different types of psychological therapy (eg, CBT, mindfulness, MI).

Studies were excluded if they included a comparison with other active lifestyle interventions unless classified as usual care. To meet objectives four and five, we looked at the usual care reported in the papers extracted from the study selection process.

### Outcomes

Where available, information on the study outcomes was collected and charted. Primary outcomes included body weight or indicator of body mass (eg, BMI), cognitive flexibility, psychological function and eating behaviours. Secondary outcomes included adverse events, quality of life, physical activity levels, stress, anxiety, depression, health behaviour, body esteem, psychological health and self-efficacy.

### Exclusion criteria

We excluded studies that were not available in English, were not full-text articles (ie, conference abstracts only), and not peer-reviewed articles (eg, magazine articles, letters, editorials, newspaper and commentary articles).

### Data synthesis and analysis

A narrative synthesis was employed to synthesise the data, prioritising and ordering data to meet the objectives of the study.^32^

### Patient and public involvement

A public advisor with more than 10 years’ experience of living with obesity provided input throughout all stages of the project and contributed to the write up of the scoping review (ie, improving the lay interpretability of the review through editing of the article).

## Results

The database searches identified 43 651 articles from four electronic databases and two trial registries. After duplicates were removed, a total of 31 177 titles and abstracts were screened for eligible studies. During the screening process, a total of 31 120 were deemed^33^ ineligible^34^ for inclusion. A total of 55 studies were extracted for full-text screening. A further two additional articles were identified for inclusion through the citation screening of the original 55 full texts. Following full text and citation screening, 31 studies were eligible for inclusion.^33^,^64^ A total of 26 articles were excluded for a range of reasons which are detailed in the PRISMA diagram (see figure 1).

### Study characteristics

Of the 31 included studies, 28 were RCTs,^3335 37^,^39 41^ and 3 were non-randomised intervention studies.^34 36 40^ Studies were conducted across 14 countries; Iran (n=8), the USA (n=4), Netherlands (n=3), Australia (n=3), Germany (n=2) and Norway (n=2), with one study from each of; Canada, Brazil, Italy, Finland, Portugal, Spain, the UK, Sweden and Thailand (see online supplemental table). The duration of the studies ranged from 2 to 51 months.^33^,^64^ Interventions were conducted mainly within clinics (n=15), but other settings included medical centres (n=11), universities (n=4) and within the community (n=1). Studies were published between 2007 and 2023, with only five studies published prior to 2012.^4059 61^,^63^ See online supplemental table for characteristics of included studies and for details of the excluded studies, refer to online supplemental material 3.

### Participant characteristics

The total number of participants within each study ranged from 31 to 1386. A total of 28 studies reported the mean age of the study sample, which ranged from 20.33 to 52.3 years.^34^,^5355 56 58 59 61^ However, only eight studies reported a mean age below 40 years of age.^3538 48^,^50 56 58 62^ Among the included studies, only seven studies reported ethnicity of the sample.^36 37 39 42 43 45 58^ Of these studies, six reported predominantly white participants (>60%).^36 39 42 43 45 58^ Most participants within the included studies were female with a total of 27 studies reporting a greater percentage female compared with male participants.^35^,^4143^ Notably, 11 studies only included female participants.^3538 40 48^,^51 56 58 59 62^

A total of 28 studies reported the mean BMI of participants which ranged from 28.2 kg/m^2^ to 53.1 kg/m^2^.^34^,^3739^ Just two studies reported socioeconomic status of the participants (both reported percentage of moderate socioeconomic status).^48 51^ All studies stipulated inclusion of participants with a BMI >25 kg/m^2^ (classified as overweight or obese).^33^,^64^

### Types of psychological interventions

The studies reported a total of 13 types of psychological interventions for weight reduction and sustained weight reduction (see online supplemental table and table 1).^33^,^64^

**Table 1:** Theoretical underpinning of the types of interventions

Type of intervention	No. of studies	Theoretical underpinning
Cognitive–behavioural therapy (CBT)	11 studies	Principles of Judith Beck’s CBT
		Principles of Christopher Fairburn’s CBT
Motivational interviewing (MI)	6 studies	Concepts of MI by Miller and Rollnick
		Self-determination theory
		Principles of the protection motivation theory
Acceptance and commitment therapy (ACT)	3 studies	Principles of Steven Hayes (ACT)
Behavioural therapy	3 studies	Concepts of social cognitive theory by Albert Bandura
		Principles of the theory of planned behaviour by Icek Azjen
Acceptance-based behavioural treatment	2 studies	Marsha Linehan’s principles of behaviour therapy
		Concepts of Alan Marlatt’s Relapse Prevention Model
Mindfulness therapy	2 studies	Jon Kabat-Zinn’s principles of cognitive therapy
Cognitive remediation therapy (CRT)	2 studies	CRT by developed by Kate Tchanturia *et al*
		Concepts of Raman *et al* (CRT)
Compassion-focused therapy (CFT)	1 study	Principles of CFT by Paul Gilbert
Tapas acupressure technique (TAT) with mental steps	1 study	Principles of Tapas Fleming’s TAT with mental steps developed by Elder *et al*
Psychotherapy (cognitive)	1 study	Principles of cognitive psychotherapy by Lisbeth Stahre
Emotional freedom technique (EFT)	1 study	Principles developed by Gary Craig (EFT)
Psychodynamic therapy	1 study	Principles developed by Jessica Yakeley and Gwen Adshead in combination with the theory of planned behaviour by Icek Azjen
Dialectical behaviour therapy (DBT)	1 study	Marsha Linehan’s principles of DBT

ACTAcceptance and commitment therapyCBTcognitive-behavioural therapyCFTCompassion-focused therapyCRTCognitive remediation therapyDBTDialectical behaviour therapyEFTEmotional freedom techniqueMIMotivational InterviewingTATTapas acupressure technique

The most common psychological therapy was CBT (n=11), followed by MI (n=6), acceptance and commitment therapy (ACT) (n=3) and behavioural therapy (n=3) (see online supplemental table). Other therapies such as acceptance-based behavioural treatment (ABT), mindfulness, cognitive remediation therapy (CRT), compassion-focused therapy (CFT), Tapas Acupressure Technique (TAT) with mental steps, psychotherapy (cognitive), emotional freedom technique (EFT), psychodynamic therapy and dialectical behaviour therapy (DBT) were reported but with less frequency (see online supplemental table). Three studies comprised multiple types of psychological therapies as they involved active comparisons between psychological interventions (see online supplemental table and table 1).

### Core intervention characteristics

The frequency of sessions within each intervention ranged from 5 to 42 sessions (average of 11 sessions). However, most studies (n=22) reported 10 or fewer sessions.^34^,^3638^ The duration of each session ranged from 20 to 180 min, but on average lasted approximately 80 min.^33^,^64^ The total duration of the interventions ranged from 225 to 5040 min, averaging 1000 min.^33^,^64^ Largely, interventions were conducted face to face (n=26), with a minority through telephone or online (n=2) and some hybrid (n=3: face to face and telephone/online) (see online supplemental table). The interventions were delivered by PhD students, research clinicians, psychotherapists, dietitians, endocrinologists, certified trainers, medical doctors, physical therapists, clinical psychologists, MI practitioners, counsellors or psychiatrists.^33^,^64^

The majority of the interventions were based on the leading theoretical foundations in their domain, as shown in table 1.

### Length of follow-up and outcomes of included studies

Most studies (n=21) included primary outcomes focused on weight reduction or changes in body mass (see online supplemental table). Those that did not, assessed either eating behaviour (including disordered eating behaviour) (n=7), cognitive flexibility (n=1), weight efficacy (n=1) or psychiatric symptomology (n=1) (see online supplemental table). Secondary outcomes included depression, waist circumference, blood pressure, quality of life, general self-efficacy activity levels, psychological health, dietary intake, health behaviours, perceived behavioural control, psychological constructs, attitudes, motivation, stress, body esteem and BMI (see online supplemental table).^57 61^

Follow-up time points ranged from 6 weeks to 36 months but studies frequently reported follow-up at 8 weeks (n=9), 6 months (n=9) or 12 months (n=15) (see online supplemental table). Of the 31 included studies, 16 measured whether participant’s weight reduction had been sustained at 12 months follow-up (see online supplemental table). Of the 16 studies which determined weight reduction at 12-month follow-up, 6 studies included CBT, 3 included behavioural therapy, 1 included MI, 1 comprised ABT, 1 involved TAT with mental steps, 1 included psychotherapy, 1 encompassed EFT and 1 involved psychodynamic therapy (see online supplemental table). The remaining 15 studies which included therapies, such as CBT (n=4), mindfulness (n=2), MI (n=4), DBT (n=1), CRT (n=2), ABT (n=1), ACT (n=2) and compassion-based therapy (n=1), did not measure outcomes following 6 months to determine if weight reduction was sustained in the longer term (see online supplemental table).

### Map of the types of usual care

**Figure 2:**
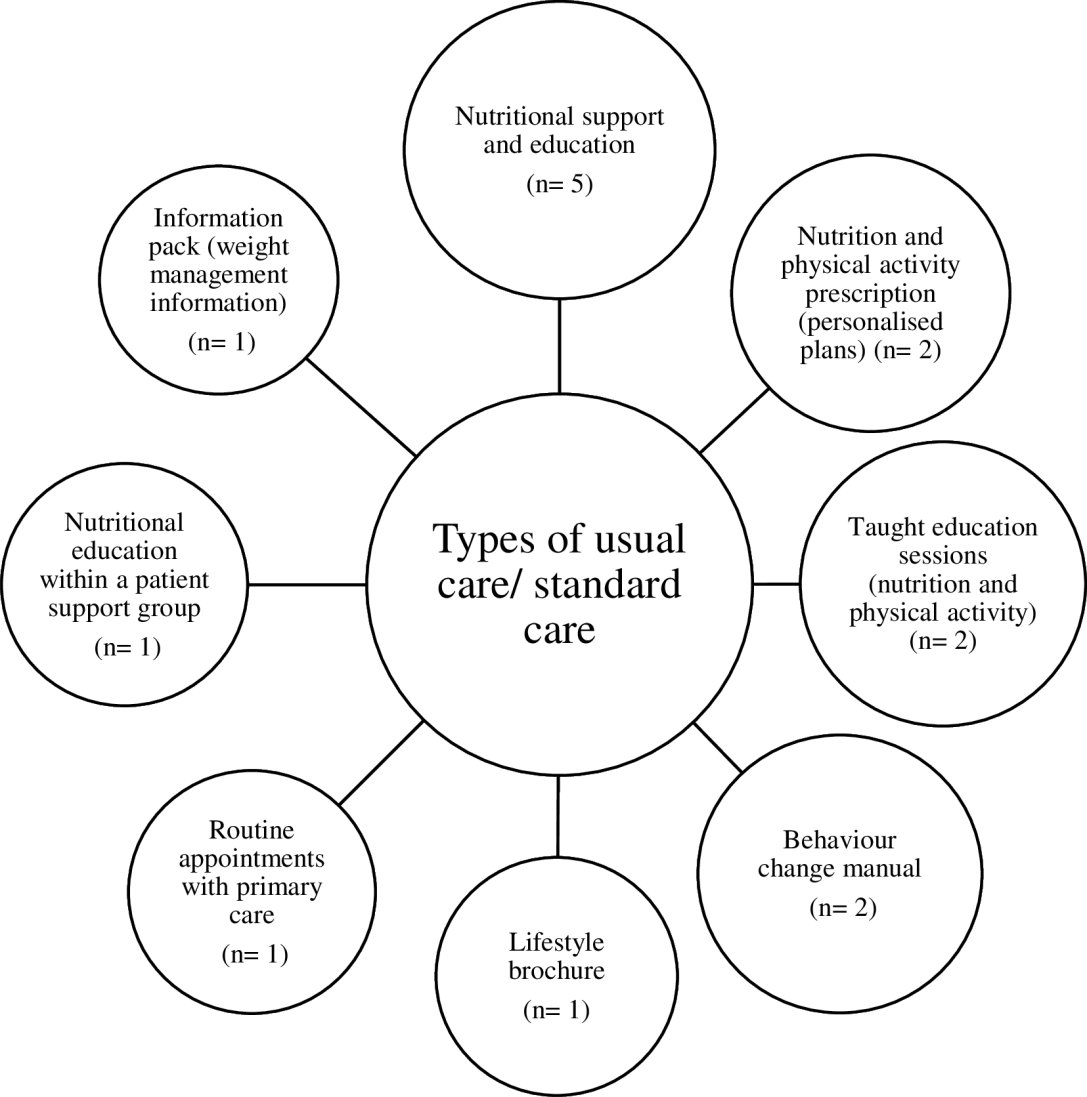
Thematic map of types of usual care for weight reduction and sustained weight reduction in adults with overweight or obesity within included studies.

Of the 31 studies, 16 included psychological interventions that were compared with usual care.^3335^,^37 39 42^ Studies used a range of terms to describe usual care such as treatment as usual, standard care and usual care. There were a total of eight types of usual care described by the included studies (see online supplemental table and figure 2). One study did not report details of the usual care which had been conducted in the control group.^44^

### Core characteristics of usual care

The 16 studies that compared an intervention to usual care reported a diverse range of characteristics related to the control group.^3335^,^37 39 42^ A total of five studies included only nutritional education and support, while another two studies included education and support on both nutritional and physical activity (see online supplemental table). A further two studies only provided a leaflet or brochure which contained information on healthy lifestyles. Two studies provided only a manual which was based on weight loss protocols and included aspects of behaviour change and coping strategies for food cravings (see online supplemental table).

Usual care was conducted in a diverse range of settings including clinics (n=3),^39 45 61^ hospitals (n=2),^53 64^ tertiary care centres (n=2),^36 44^ a healthcare centre^35^ and a research centre.^33^ Seven studies did not report the setting of the usual care.^3842 43 49^,^51 57^ Usual care was typically delivered by a primary care practitioner (n=6; eg, doctors, nurses)^33 36 44 48 62 64^ or doctoral student (n=1),^42^ although most studies did not report the provider (n=8).^3539 42^,^45 50 51^ The frequency of sessions within usual care ranged from one to six sessions, averaging three sessions.^3335^,^37 39 42^ The duration of usual care ranged from 2 weeks to 18 months but on average continued for approximately 6 months.^3335^,^37 39 42^

### Gaps within the evidence

There were several gaps within the existing evidence identified by the findings of this review. First, there is a dearth of literature relating to younger adults (under 40 years of age) with overweight and obesity who have undertaken psychological therapies as a means of weight reduction and sustained weight reduction. Second, the ethnic diversity of the current evidence appears narrow, with most existing studies involving predominantly white participants (>60%). The limited ethnic representation increases concerns relating to the generalisability of findings to non-white ethnic groups, which is important given that research suggests that people from ethnic minorities (eg, Bangladeshi, Pakistani, black African and black Caribbean populations) may be at greater risk of obesity compared with Caucasians^65 66^ Third, there is a lack of literature highlighting the impact of how psychological therapies may influence weight reduction in adults with low socioeconomic status. Fourth, few studies have explored digital health technologies (eg, mobile applications, virtual meeting software) as a means of delivering psychological therapy interventions for adults with overweight or obesity. Finally, there is a notable absence of studies exploring therapy approaches such as counselling (integrative, eclectic), guided self-help, interpersonal therapy, cognitive analytic therapy, rational emotive behavioural therapy, exposure therapy, humanistic therapies, existential therapy, artistic therapies, music therapy, psychodrama and systemic therapy. As a consequence, little is known about the potential effectiveness of these psychological therapy interventions as a means of achieving weight reduction and sustained weight reduction in adults with overweight and obesity.

## Discussion

This review provides an up-to-date synthesis of existing therapy interventions used to promote weight reduction in adults with overweight and obesity.^20 23 67^ This scoping review identified the types and characteristics of psychological therapy interventions and usual care for weight reduction and sustained weight reduction in adults with overweight and obesity. A total of 13 types of psychological interventions for weight reduction and sustained weight reduction in adults with overweight and obesity were identified by the review. These interventions were primarily delivered face to face by healthcare professionals, and on average, composed of 11 sessions lasting 80 min each. In addition, eight types of usual care were identified, typically involving education and training on nutrition and physical activity. Individuals receiving usual care received fewer appointments from their primary healthcare provider compared with those receiving psychological therapy interventions.

The findings of this scoping review expand on older reviews that have documented the types of psychological therapy interventions for adults with overweight and obesity.^20 23 67 68^ In addition to interventions such as CBT, DBT, behaviour therapy, psychotherapy, MI and mindfulness which have been identified by older reviews,^20 67 68^ this scoping review identified four additional psychological therapy interventions that have emerged within publications over the past 5 years (ie, ACT, EFT, CRT and compassion-based therapy).^20 23 68^ Although these interventions may be novel in this field of research, our review shows that the evidence is limited by small sample sizes and short-term follow-up. In addition, the current findings also highlight that there is some uncertainty about which psychological interventions are effective, which therapy types may result in the greatest weight reduction, and which have sufficient certainty of evidence for recommendations into clinical practice.

This review identified several types of usual care that were employed as comparators for psychological therapy interventions across the included studies. Typically, usual care composed of information and support to improve patient nutritional intake, although in addition, several usual care groups also received guidance on increasing their levels of physical activity (through information packs and leaflets). These components are consistent with the guidance from key organisations (ie, National Institute for Health and Care Excellence and WHO) and health services (ie, National Health Service, NHS), which advocate that the main treatment for overweight and obesity should focus on increasing energy expenditure (physical activity) and reducing energy intake (dietary modifications).^3 69 70^ This review shows that usual care interventions offered by primary care practitioners generally do not include psychological components to support individuals with weight reduction. Given the complex psychological, emotional and behavioural factors that likely underpin excess weight, usual care which does not incorporate a psychological component may not be wholly effective at reducing the prevalence of population overweight and obesity.^16 71^ With that said, the inclusion of psychological therapy interventions into usual care practice may require additional resources and investment.^52^ To determine whether these interventions could be delivered at scale within the NHS (through incorporation into usual care), further research in the form of an economic evaluation is needed.

This review highlighted several key evidence gaps which are consistent with findings already available in the literature that have highlighted an under-representation of younger adults and adults with obesity from different socioeconomic status backgrounds.^26 66^ Further research is required to improve understanding of how psychological therapies may impact weight reduction in these groups. In addition, an up-to-date comprehensive systematic review is needed to determine the effectiveness of the different types of psychological interventions for weight reduction (in adults with overweight and obesity), given that the most recent comprehensive systematic review in this area was last published in 2005.^20^ Further research is also needed to explore the effectiveness of psychological interventions which integrate digital technologies as a means to achieve weight reduction in adults with overweight and obesity. With the recent rise in smartphone applications, wearable devices, virtual reality and online platforms, digital health technology holds the potential to enhance the effectiveness of psychological therapies and reduce waiting times for patients.^72 73^ This potential has been evidenced by previous reviews that show psychological interventions which incorporate digital technology (eg, computer-assisted therapy, smartphone apps and wearable technologies), can be effective for the treatment of important clinical outcomes (eg, depression and anxiety).^74 75^

### Strengths and limitations

One of the notable strengths of this scoping review is the methodology employed. Given the objectives of this review, using a scoping review methodology allowed for more expansive inclusion criteria (compared with a systematic review methodology), ensuring all relevant studies relating to psychological therapy interventions for adults with overweight and obesity could be included. The methodology ensured that specific gaps within the existing knowledge base (which are useful in shaping future research initiatives) were identified. In contrast, a systematic review on the same topic would have likely restricted the inclusion criteria to only include higher-quality evidence (eg, RCTs) and excluded studies examining different types of therapy or those that did not report specific outcomes. However, an important limitation of this scoping review is that it did not include complex multicomponent interventions or studies comparing psychological therapy interventions to active nutritional and/or physical activity interventions, and as such there is a degree of uncertainty as to whether there may be other psychological therapy interventions for adults with overweight and obesity not described within this scoping review. A further limitation of this review is that it only included RCT and non-randomised intervention studies. Consequently, studies with alternative designs would not have been included, despite the potential that they may have been relevant to the findings of this review. An additional limitation is that the eligibility criteria regarding psychological therapy interventions may have limited the inclusion of studies relevant to mapping the types of usual care for weight reduction and sustained weight reduction in adults with overweight or obesity. As a consequence, some types of usual care may not have been included in the findings of this review. A final limitation of this study relates to the search strategy, in which there was a notable reduction in studies from 31 177 initial database hits to a total of 31 included studies. This reduction deviates from the ratio of database hits to included studies observed in other scoping studies.^76 77^ This substantial drop in numbers raises concerns regarding the efficiency and specificity of our search strategy which could have been prevented with additional search limits (eg, adult age group restrictions, along with refinement of the search terms). Revisions to the search strategy would have likely aligned our search numbers with those seen in other reviews, improving the efficiency of the screening process.

## Conclusion

This review identified a total of 13 types of psychological therapy interventions which promoted weight reduction in adults with overweight and obesity. The most common interventions identified by this review were CBT and MI. Psychological interventions were predominantly delivered in multiple face-to-face sessions by healthcare professionals (based in different settings), with participants receiving an average of a thousand minutes of therapy. The primary outcomes of the studies focused on weight reduction and changes in body mass, while secondary outcomes often measured depression, waist circumference, quality of life and health behaviours. In contrast, eight types of usual care were identified which typically involved educating and training focused on nutrition and physical activity. Usual care was largely delivered by primary care practitioners through multiple appointments across several months. The findings highlight several gaps within the existing literature, largely due to a lack of evidence relating to adults with low socioeconomic status, non-white participants and individuals under 40 years of age. Further research is required in the form of high-quality RCTs to explore how psychological therapies may impact weight reduction in these groups. Further research is also needed in the form of a comprehensive systematic review to determine the effectiveness of the different types of psychological interventions for weight reduction in adults with overweight and obesity.

## supplementary material

10.1136/bmjopen-2023-082973 online supplemental file 1

10.1136/bmjopen-2023-082973 online supplemental file 2

10.1136/bmjopen-2023-082973 online supplemental file 3

10.1136/bmjopen-2023-082973 online supplemental file 4

## Data Availability

Data sharing is not applicable as no datasets were generated and/or analysed for this study.
