# Supplementary material for: Psychological interventions for weight reduction and sustained weight reduction in adults with overweight and obesity: a scoping review
Source: BMJ Open. 2024 Dec 2;14(12):e082973. doi: 10.1136/bmjopen-2023-082973 (PMC11624810; doi:10.1136/bmjopen-2023-082973)
Supplement: online supplemental file 3 [file bmjopen-14-12-s003.pdf]

Supplementary material 3

Excluded studies table.

Excluded study (Citation)	Reasons for exclusion
Baur, J., Krohmer, K., Naumann, E., & Svaldi, J. (2022). Efficacy and mechanisms of change in exposure-based and cognitive stand-alone body image interventions in women with overweight and obesity. Behaviour research and therapy, 159, 104210.	Exclude -Wrong designation - not weight loss focused on body dissatisfaction and interview-based shape concerns
Blevins, N. (2009). Mindfulness meditation as an intervention for body image and weight management in college women: a pilot study. Dissertation Abstracts International: Section B: The Sciences and Engineering. 69.	Exclude -Abstract, no full text available
Cattivelli, R & Guerrini U, Anna M, Gian , Riboni, F & Pietrabissa, G, Musetti, Alessandro & Franceschini, Christian & Varallo, G, Spatola, C, Giusti, E. Castelnuovo, G, Molinari, E. (2021). ACTonFood. Acceptance and Commitment Therapy-Based Group Treatment Compared to Cognitive Behavioral Therapy-Based Group Treatment for Weight Loss Maintenance: An Individually Randomized Group Treatment Trial. International Journal of Environmental Research and Public Health. 18. 9558.	Exclude - Wrong intervention - included exercise training sessions in combination with psychological therapy and also had an active comparator.
Castelnuovo, G., Manzoni, G. M., Villa, V., Cesa, G. L., Pietrabissa, G., & Molinari, E. (2011). The STRATOB study: design of a randomized controlled clinical trial of Cognitive Behavioral Therapy and Brief Strategic Therapy with telecare in patients with obesity and binge-eating disorder referred to residential nutritional rehabilitation. Trials, 12, 114.	Exclude - Protocol only
Cooney, L. G., Milman, L. W., Hantsoo, L., Kornfield, S., Sammel, M. D., Allison, K. C., Epperson, C. N., & Dokras, A. (2018). Cognitive-behavioral therapy improves weight loss and quality of life in women with polycystic ovary syndrome: a pilot randomized clinical trial. Fertility and sterility, 110(1), 161–171.e1.	Excude - Wrong comparator - nutritional active control
Dehrouyeh, S., Olia Emadian, S., Tavakoli, N., & Tavakoli, N. (2022). The Effectiveness of Dialectical Behavior Therapy on Emotion Regulation, Food Cravings and Body Mass Index in Obese Female Students. Razavi International Journal of Medicine, 10(3), 9-15.	Exclude - Wrong population - high school/ adolescent students under 18 years of age
Perri, M.G., Limacher, M.C., von Castel-Roberts, K., Daniels, M.J., Durning, P.E., Janicke, D.M., Bobroff, L.B., Radcliff, T.A., Milsom, V.A., Kim, C. and Martin, A.D. (2014), Comparative effectiveness of three doses of weight-loss counselling. Obesity journal symposium.	Exclude -Abstract, no full text available
Preuss, H. (2013). Evaluation of group psychotherapies to improve the self-regulation of overweight/obese people. German Clinical Trials Register: DRKS-ID: DRKS00005250	Exclude - Protocol only
Snjezana P. Role of cognitive-behavioral therapy in the treatment of obesity. Endocrine Abstracts (2017) 49 EP712.	Exclude -Abstract, no full text available

Giada, P. (2018). Group Motivation-Focused Interventions for Patients With Obesity and Binge Eating Disorder. Frontiers in Psychology 9.	Exclude -Abstract, no full text available
Kachooei M, Hasani J, Taghizadeh F. Comparison of effectiveness of cognitive reappraisal and mindfulness based interventions in modulating body mass index (BMI), Food craving and binge eating in overweight women. Feyz 2021; 25(3): 951-62	Exclude - Protocol only
Babakhan, K. (2019). Comparison of Effectiveness of Integrative Group Psychotherapy and Cognitive-Behavioral Stress Management Therapy Group on the Body Image, Somatic Symptom, Mindful Eating and The Satisfaction With Therapy and Therapist in Obese People. WHO clinical Trials registry Irct20181214041964N1, 2019	Exclude - Protocol only
Kebriti, H. (2020). Effect of mindfulness-based stress management therapy on the emotion regulation ,anxiety ,depression and food addiction in obese people. WHO clinical Trials registry: IRCT20190804044436N1.	Exclude - Protocol only
Jandaghian, M. (2020). The effect of dialectical behavior therapy on psychological symptoms and Body measurement indices. WHO clinical Trials registry: Irct20190827044626N1.	Exclude - Protocol only
Rostami, M. (2020) The effectiveness of cognitive Hypnotherapy on the coping self-efficacy, executive functions, Cognitive Emotional Regulation, mental health, Food Craving and weight loss of Women with Obesity. WHO clinical Trials registry: Irct20200622047889N1.	Exclude - Protocol only
Zergani, M. (2020) Effect of mindfulness training on weight loss. WHO clinical Trials registry: Irct20200919048767N1.	Exclude - Protocol only
Iturbe I, Pereda-Pereda E, Echeburúa E, Maiz E. (2019) The Effectiveness of an Acceptance and Commitment Therapy and Mindfulness Group Intervention for Enhancing the Psychological and Physical Well-Being of Adults with Overweight or Obesity Seeking Treatment: The Mind&Life Randomized Control Trial Study Protocol. International Journal of Environmental Research and Public Health. 20(4).	Exclude -Protocol only
Iturbe I, Pereda-Pereda E, Echeburúa E, Maiz E. (2021) The Effectiveness of an Acceptance and Commitment Therapy and Mindfulness Group Intervention for Enhancing the Psychological and Physical Well-Being of Adults with Overweight or Obesity Seeking Treatment: The Mind&Life Randomized Control Trial Study Protocol. International Journal of Environmental Research and Public Health, 18(9):4396.	Exclude -Abstract, no full text available
Jackson, J. B., Pietrabissa, G., Rossi, A., Manzoni, G. M., & Castelnuovo, G. (2018). Brief strategic therapy and cognitive behavioral therapy for women with binge eating disorder and comorbid obesity: A randomized clinical trial one-year follow-up.Journal of Consulting and Clinical Psychology, 86(8), 688–701.	Excude - Wrong intervention - included exercise training sessions and Mediterranean diet in combination with psychological therapy.]
Kanaya A. M. (2012). ACP Journal Club. Enhanced brief lifestyle counseling for obesity was better than usual care for weight loss at 2 years. Annals of internal medicine, 156(6), JC3–JC10.	Exclude - No Full Text.
Levin, M. E., Petersen, J. M., Durward, C., Bingeman, B., Davis, E., Nelson, C., & Cromwell, S. (2021). A randomized controlled trial of online acceptance and commitment therapy to improve diet and physical activity among adults who are overweight/obese. Translational Behavioral Medicine, 11(6), 1216-1225.	Exclude - Wrong intervention - solely online - no psychological input

Mallorqui-Bague, Nuria; Lozano-Madrid, Maria; Vintro-Alcaraz, Cristina; Forcano, Laura; Diaz-Lopez, Andres; Galera, Ana; Fernandez-Carrion, Rebeca; Gr (2021). Effects of a psychosocial intervention at one-year follow-up in a PREDIMED-plus sample with obesity and metabolic syndrome. Scientific Reports, 11(1)	Exclude - Wrong intervention - included receiving a diet in combination with psychological
Moritz, S., Göritz, A.S., Schmotz, S. et al. (2019). Imaginal retraining decreases craving for high-calorie food in overweight and obese women: A randomized controlled trial. Transl Psychiatry 9, 319.	Exclude - Wrong intervention - solely online - no psychological input by a person at any point
Nourizadeh, R., Azami, S., Farshbaf-Khalili, A., & Mehrabi, E. (2020). The Effect of Motivational Interviewing on Women with Overweight and Obesity Before Conception. Journal of nutrition education and behavior, 52(9), 859–866.	Exclude - Wrong comparator - active nutritional intervention
Perri, M. (2014) Effects of Behavioral Treatment on Long-Term Weight Loss: Lessons Learned From the Look AHEAD Trial. Obesity.	Exclude – Abstract only
Raman, J., Tchanturia, K., & Hay, P. (oral presentation, May 2016). Manualised cognitive remediation therapy for adult obesity: A randomised controlled trial. World Congress Behavioural and Cognitive Therapies WCBCT 2016	Exclude -Abstract, no full text available
Wadden, T. A., Hollander, P., Klein, S., Niswender, K., Woo, V., Hale, P. M., & Aronne, L. (2014). Weight maintenance and additional weight loss with liraglutide after low-calorie-diet-induced weight loss: The SCALE Maintenance randomized study. International journal of obesity 2005, 39(1), 187.	Exclude - Wrong intervention - included receiving a diet (meal replacements) in combination with psychological
