# Supplementary material for: Psychological interventions for weight reduction and sustained weight reduction in adults with overweight and obesity: a scoping review
Source: BMJ Open. 2024 Dec 2;14(12):e082973. doi: 10.1136/bmjopen-2023-082973 (PMC11624810; doi:10.1136/bmjopen-2023-082973)
Supplement: online supplemental file 4 [file bmjopen-14-12-s004.pdf]

Table 1. Characteristics of included studies.

Author(s) and year	Study location	Number of participants (n)	Type of psychological intervention*	Mode	Number of sessions – (total duration)	Comparison	Type of usual care/ intervention comparison	Primary outcomes	Secondary outcomes	Follow-up time points
Abedishargh et al, 2021	Iran	90	Cognitive Behavioural Therapy (CBT)	Hybrid	42 (5040 minutes)	Usual care	Nutritional support and education	Body mass	Stress, anxiety, and depression	6 weeks
Asadollahi et al, 2015	Iran	60	Mindfulness	Face-to-face	8 (960 minutes)	No intervention	No treatment	Body mass	Eating behaviour	8 weeks
Azami et al, 2020	Iran	70	Motivational interviewing (MI)	Face-to-face	6 (540 minutes)	Usual care	Not reported	Body mass	Energy intake	2 & 6 months
Barnes et al, 2017	USA	89	Motivational interviewing (MI)	Face-to-face	5 (140 minutes)	Usual care	Routine appointments with primary care provider	Body mass	Motivation, depression, blood pressure and eating behaviour	3 & 12 months
Berk et al, 2018	Netherlands	158	Cognitive Behavioural Therapy (CBT) (group)	Face-to-face	15 (Not reported)	Usual care	Nutritional support and education	Body mass	Waist circumference, blood pressure, cholesterol, depression, anxiety, self-esteem, quality of life, fatigue, and physical activity	2, 3, 12 & 24 months
Cancian et al, 2019	Brazil	31	Dialectical Behaviour Therapy (DBT)	Face-to-face	10 (1200 minutes)	No intervention	No treatment	Eating behaviour	Emotion regulation and psychiatric symptoms	8 weeks
Cassin et al, 2016	Canada	47	Cognitive Behavioural Therapy (CBT)	Online/ Telephone	6 (270 minutes)	Usual care	Nutrition education within a monthly support group	Eating behaviour	Psychosocial functioning	7 weeks
Cresci et al, 2007	Italy	141	Cognitive Behavioural Therapy (CBT)	Face-to-face	10 (900 minutes)	Active psychological intervention	CBT	Weight loss	Physical activity, dietary intake, and drug use	3, 6 & 12, 36 months
Elder et al, 2012	USA	285	Tapas Acupressure Technique (TAT) with mental steps	Face-to-face	9 (270 minutes)	Active psychological intervention	Social support intervention	Weight loss	Personal health, perceived stress, insomnia, and quality of life	6 & 12 month
Forman et al, 2013	USA	128	Acceptance-based behavioural treatment (ABT)	Face-to-face	30 (2250 minutes)	Usual care	Behaviour change manual	Body mass	Quality of life, food habits, depression, and food cravings	10, 20, 40 weeks, & 6 month
Forman et al, 2016	USA	190	Acceptance-based behavioural treatment (ABT)	Face-to-face	16 (1200 minutes)	Usual care	Behaviour change manual	Weight loss	Health behaviours, food cravings and depression	12 months
Gade et al, 2015	Norway	80	Cognitive Behavioural Therapy (CBT)	Face-to-face	10 (Not reported)	Usual care	Not reported	Body mass	Eating behaviour and depression	12 months

Hilbert et al, 2022	Germany	270	Cognitive remediation therapy (CRT)	Face-to-face	8 (960 minutes)	No intervention	No intervention	Body mass	BMI, executive function, weight management behaviours, self-efficacy, mental health, quality of life, physical health, hip and waist circumference, blood pressure, bioelectrical impedance, and subscapularis skinfolds	6 months
Hjelmesaeth et al, 2015	Norway	61	CBT focusing on self-monitoring	Hybrid	11 (Not reported)	Usual care	Nutritional support and education	Eating behaviour	Depression and BMI	12 & 48 months
Jamshidi et al, 2023	Iran	60	Cognitive Behavioural Therapy (CBT)	Face-to-face	8 (720 minutes)	No intervention	No intervention	Weight Efficacy	BMI, waist circumference and body fat percentage	8 weeks
Jarvela-Reijone et al, 2018	Finland	219	Acceptance and commitment therapy (ACT)	Face-to-face	6 (540 minutes)	No intervention	No intervention	Eating behaviour	Stress, and general health	8 & 36 weeks
Mirkarimi et al, 2015	Iran	150	Motivational Interviewing (MI)	Face-to-face	5 (225-300 minutes)	Usual care	Nutritional support and education	Body weight	Protection motivation theory constructs	8 weeks
Mirkarimi et al, 2017	Iran	150	Motivational Interviewing (MI)	Face-to-face	5 – (225-300 minutes)	Usual care	Nutritional support and education	Body weight	Protection motivation theory constructs	8 weeks
Palmiera et al, 2017	Portugal	73	Mindfulness, ACT, and compassion-based therapy	Face-to-face	12 (1800 minutes)	Usual care	Nutrition & physical activity prescription plans	Eating behaviour	BMI, quality of life, activity levels, cholesterol, psychological function, and weight stigma	14 weeks
Paul et al, 2021	Netherlands	128	Cognitive Behavioural Therapy (CBT)	Face-to-face	10 (450 minutes)	Usual care	Routine appointments with primary care provider	Body mass	Eating behaviour, eating disorders, depression, quality of life, and overall psychological health	10 weeks & 12 months
Porca et al, 2021	Spain	437	Group behavioural therapy	Face-to-face	6 (360 minutes)	Usual care	Nutrition & physical activity prescription plans	Body mass	Change of lifestyle adherence and lifestyle habits	12 months
Raman et al, 2018	Australia	80	Cognitive remediation therapy for obesity (CRT)	Face-to-face	8 (360 minutes)	No intervention	No intervention	Cognitive flexibility	Weight change, binge eating, BMI, depression, and quality of life	8 weeks & 3 months
Rieger et al, 2017	Australia	201	CBT with support person (CBT)	Face-to-face	26 – (2340 minutes)	Active psychological intervention	Not reported	Body mass	Self-efficacy, quality of life, treatment acceptability, body esteem and binge eating	12 & 24 months
Saffari et al, 2014	Iran	327	Motivational Interviewing (MI)	Face-to-face	5 (300 minutes)	No intervention	No intervention	Eating behaviour	BMI and metabolic assessment	12 months
Shemirani et al, 2021	Iran	45	CBT and ACT	Face-to-face	9 (810 minutes)	No intervention	No intervention	Eating behaviour	Health promoting behaviour	8 weeks
Simpson et al, 2015	UK	166	Motivational Interviewing (MI)	Hybrid	15 (540 minutes)	Usual care	Information pack	BMI	Waist and hip circumference, physical activity levels, diet, quality of life, alcohol	8 weeks

									consumption, eating behaviour and dietary intake	
Stahre et al, 2005	Sweden	105	Psychotherapy (cognitive)	Face-to-face	10 (1800 minutes)	No intervention	No intervention	Weight loss	None	6, 12 & 18 months
Stapleton et al, 2017	Australia	178	Emotional Freedom Technique and CBT	Face-to-face	8 (960 minutes)	No intervention	No intervention	Psychiatric symptom-ology	BMI and general health	6 and 12 months
Van Wier et al, 2009	Netherlands	1,386	Behaviour therapy	Online/ Phone	10 (Not reported)	Usual care	Lifestyle brochures	Body mass	Perceived health, empowerment, self-efficacy concerning weight control, physical activity and eating habits, work performance, waist circumference, sum of skin folds, blood pressure, total blood cholesterol level and fitness	6, 12, 18 & 24 months
Waleekhachonloet et al, 2007	Thailand	132	Behaviour therapy (group and individual)	Face-to-face	5 (360-480 minutes)	Active psychological intervention	Behavioural therapy	Weight loss	BMI, dietary intake, healthy dieting behaviour, perceived behavioural control, attitude, and subjective norms	3, 6, & 12 months
Wiltink et al, 2007	Germany	267	Psychodynamic therapy	Face-to-face	Not reported	No intervention	Not reported	Body mass	Eating behaviour, physical complaints, interpersonal problems, locus of control, general self-efficacy, satisfaction with weight and treatment satisfaction	36 months
