# Supplementary material for: Psychological interventions for weight reduction and sustained weight reduction in adults with overweight and obesity: a scoping review
Source: BMJ Open. 2024 Dec 2;14(12):e082973. doi: 10.1136/bmjopen-2023-082973 (PMC11624810; doi:10.1136/bmjopen-2023-082973)
Supplement: Supplementary file 5 [file bmjopen-2023-082973.reviewer_comments.pdf]

PEER REVIEW HISTORY

BMJ Open publishes all reviews undertaken for accepted manuscripts. Reviewers are asked to complete a checklist review form and are provided with free text boxes to elaborate on their assessment. These free text comments are reproduced below.

ARTICLE DETAILS

Title (Provisional)

Psychological interventions for weight reduction and sustained weight reduction in adults with overweight and obesity: a scoping review

Authors

Hamer, Oliver; Kuroski, Jennifer A; Bray, Emma P; Harris, Cath; Blundell, Amy; Schneider, Emma; Watkins, Caroline

---

VERSION 1 - REVIEW

**Reviewer**	**1**
**Name**	**De Poli, Chiara**
**Affiliation**	**London School of Economics and Political Science, Management**
**Date**	**16-Jan-2024**
**COI**	**none**

Thanks for the opportunity to read the article "Psychological interventions for weight reduction and sustained weight reduction in adults with overweight and obesity: a scoping review". The article aims to map the evidence available on the types and characteristics of psychological interventions for weight reduction and sustained weight reduction in adults with overweight or obesity. The topic is certainly relevant and of interest. The review has been conducted in a rigorous way and reported clearly. int he document attached I offer some comments for the consideration of the Authors.

Lay summary

- Some wording could be tweaked to improve accessibility of the summary to a genuinely lay audience. Terms such as sample size, gaps in the evidence, database, inclusion, and exclusion criteria, are not accessible to a non specialist audience. I would suggest revising the summary taking this comment into account

Abstract

Objectives: “psychological interventions and usual care” – this could be clarified as “psychological interventions vs usual care”?

Results – “thirteen psychological interventions for weight reduction in adults with overweight and obesity were identified” – should this read as “adults with overweight OR obesity”?

Introduction

- Globally, the prevalence of obesity has risen to approximately 650 million (adults ≥18 years of age) - Could this sentence being reworded as “Globally, the prevalence of obesity among the adult population (ie, individuals aged 18 and above) has risen to approximately 650 million” – or something to this effect
- Can you also provide a definition for overweight and obesity
- Overweight and obesity have a multifaceted aetiology, with factors such as genetics, biological influences, social components, medical conditions, stress, lifestyle factors, economic aspects, and environmental factors contributing to prevalence. – can these factors be represented in a more organic way and using some criteria, eg bio-medical vs socio-cultural, or individual- vs environmental-factors?
- “Over the past two decades, several interventions have been developed to tackle overweight and obesity which have largely focused on lifestyle modifications 7,” – there has been a growing emphasis also on population-based interventions to tackle overweight and obesity, such as various forms of sugar taxes, regulation of advertising, reform of licensing of food outlets etc. I think it is important to mention these alongside individual based interventions targeting lifestyle change
- “While increasing energy expenditure and reducing dietary intake are critical for weight loss (in most cases), they often ignore the complex psychological, emotional, and behavioural factors that underpin overweight and obesity” – I would argue that also broader environmental factors can contribute to overweight and obesity – eg lack of safe parks and public areas for physical activity, lack of public transport that could be used as an alternative to driving, exposure to advertising promoting unhealthy food, affordability of healthy food options, etcetc.
- p.3 L.46 “the psychological factors that contribute to overweight and obesity” – can you spell out these psychological factors. And what is the causal chain between these and overweight or obesity?
- p.3 l.50 – I would avoid “etc”
- p.3 l.55 – what is the difference between weight reduction and weight management
- p.3 l.55-56 “In recent years, research in the field of weight reduction and weight management has rapidly evolved with a particular focus on psychological interventions” – can we explain why psychological interventions have been implemented more and more?
- l.38 and 55, the two pars start in the same way “In recent years” – can you avoid the repetition?

- p.3 l.58-59 “to assess the effectiveness of psychological interventions for weight reduction in adults with weight concerns” – what is meant by weight concern? Is the focus only weight reduction or also weight management mentioned above?
- p.4 “However, these reviews are limited by population (including only adults with specific medical conditions), type of interventions (including only one type of psychological therapy intervention), or being substantially outdated” – can you spell out the limitations of the reviews mentioned here and provide references for each. Eg what is meant by being substantially outdated? Why you think it is problematic that a review focused only on one type of psychological therapy intervention? What populations have been covered by previous reviews?

Aim

- What is the difference between weight reduction and sustained weight reduction? Can you define both?

Objectives

- Objectives 1-3 are clear and consistent with the aim of the review (provided a definition of weight reduction and sustained weight reduction and of “adults”), I am not clear why among the objectives of the review three focus on usual care for weight reduction and sustained weight reduction (see objectives 4-6). Can you please clarify/justify, since the scoping review does not map the results of the trials?

Search strategy

- “We searched the above databases and trials registries from their inception to April 2023 (present)” – what was the added value of searching databases from inception? Were the definitions of overweight and obesity the same over this period? What was the state of the art of psychological interventions in the 50s compared to 2010s?
- Can you justify why you have decide to include only Randomised controlled trials (RCTs) or non-randomised comparative intervention studies?
- Can you specify that you consider for inclusion only interventions delivered by a professional – so it is clear that you are excluding those where a professional is not involved (eg interventions delivered via an app)

Types of interventions

- “Individual and group therapies were included” – Can this be reworded as “Interventions delivered one-to-one or in group setting were included”
- How did you deal with trials with a multi-pronged approach, e.g trials that combined a lifestyle intervention (eg providing advice on healthy diet, the importance of physical activity) and/or medications and/or a psychological intervention and/or multiple/integrated psychological interventions?

- For the comparison,
    - how was no intervention defined? No psychological intervention? No intervention of any type?

Outcomes

- No psychological outcomes are listed here, which seems surprising (eg measures of self-efficacy, etc). can you please justify.
- Table 1 though list secondary outcomes which are also psychological in nature. Can you please clarify in this section of the paper what outcomes intended to report?

Data synthesis and analysis

- Can you provide details about Synthesis WIthout Meta-analysis in systematic reviews (SWiM)? Why did you think it was relevant for a scoping review?

Participant characteristics

- Did you notice any differences in the definition of overweight and obesity used in each study/over time/in different countries?

Gaps within the evidence

- You observe that "there is a notable absence of studies exploring therapy approaches such as counselling (integrative, eclectic), guided self-help, interpersonal therapy, cognitive analytic therapy, rational emotive behavioural therapy, exposure therapy, humanistic therapies, existential therapy, artistic therapies, music therapy, psychodrama, and systemic therapy" – did your search strings aim to identify such approaches/were your search strings broad enough to allow you identify studies using such approaches?

Discussion

- I am not entirely sure about the aim of the comparison between psychological interventions vs usual care, since the results of the comparison are presented in a descriptive way (eg "Individuals receiving usual care received fewer appointments from their primary healthcare provider compared to those receiving psychological therapy interventions"), but no information is provided about the outcomes of usual care vs psychological interventions. Would not it be interesting to know what outcomes are achieved by each intervention arm or the difference between each intervention arm? If you think this is beyond the aim of a scoping review, why did you focus the review also on the comparators?
- The Discussion repeats some content included in the Gaps within the evidence – can this be streamlined?
- Besides the clinical relevance of the interventions, do you want to comment on the feasibility of delivering **at scale** the interventions that you reviewed? Using the English context as an example, would the health care system have capacity and resources for delivering these interventions at scale?

Strengths and limitations

- I think it should be explicitly acknowledged that the scope of the review excluded studies not designed as an RCT or a non-randomised intervention study. Studies such as those using qualitative research methods that aim to explore e.g. the feasibility of delivering psychological interventions in an overweight/obese population or the acceptability of such interventions among overweight/obese individuals and across individuals from different socio-demographic backgrounds (among whom maybe obesity is highly stigmatised) or studies that explain the take up of psychological interventions in an overweight/obese population (eg why people drop out from such interventions) were not included, despite the relevance of the evidence they can provide. Notably, all these aspects affect the effectiveness, uptake, etc of interventions tested out in RCTs.

Conclusion

- Psychological interventions were predominantly delivered in multiple face-to-face settings by healthcare professionals – can this be reworded as "Psychological interventions were predominantly delivered in multiple face-to-face encounters by healthcare professionals based in different settings" – or something to this effect

---

Reviewer 2

Name Hazlehurst, Jonathan

Affiliation University of Birmingham, WHO Collaborating Centre for Global Women's Health, Institute of Metabolism and Systems Research,

Date 30-Jan-2024

COI None

I read this scoping review with interest. There are a number of significant queries that will need to be addressed. That said this is an important piece of work and given the right responses could represent a significant contribution to the literature.

There are a few small grammatical errors in the otherwise thoughtful and well written lay summary that will no doubt be picked up at the editorial stage. Eg will also identifies gaps

The abstract in its current form is not adequate and does not appropriately confirm with the PRISMA-ScR checklist. It needs to be re written. The authors may find this scoping review published in BMJ Open a useful reference for re writing the abstract From screen time to the digital level of analysis: a scoping review of measures for digital media use in children and adolescents | BMJ Open In particular in the results of the abstract it was not clear whether the authors found 13 different psychological interventions of 13 in total.

The introduction whilst otherwise well written is lacking a section on weight loss medications and bariatric surgery to better set the scene for the importance of psychological treatment.

The search terms used, whilst appropriate for the bulk of the work, could not possibly hope to address outcome 4 and 5 given the search does not identify all “usual care” only studies incorporating a psychological treatment. This is a serious concern, especially given the published protocol paper in the same journal. If I am wrong and have missed something it should be addressed.

The rest of the methods seems appropriate and is in line with the groups published protocol paper. In the results I see that 2 full text were excluded for “wrong comparator” yet there is not information given in inclusion or exclusion section about the comparator required. This needs to be justified.

The PRIMSA flow chart includes* and ** with no legend for its interpretation

Can the authors confirm that in Table 1 where an intervention is not specifically labelled as group it should be assumed to be individual

I have no further comments

**Reviewer**	**3**
**Name**	**Mey, Jacob**
**Affiliation**	**Pennington Biomedical Research Center**
**Date**	**09-Feb-2024**
**COI**	**n/a**

I read with interest the scoping review by Hamer et al on Psychological interventions for weight and maintenance in adults. I was particularly appreciative of the inclusion of a public advisor and the wide-spread use of person-first language (Notably, the article does still needs additional editing/double checking for person-first language, as instances of inappropriate use still remain e.g., Page 17 of 30, line 58 “who are … obese” should be “who have … obesity”). Within the context of the actual articles included, the authors effectively addressed their stated objectives in concise and comprehensive fashion. Still, I have several concerns about this search and selection of articles given the provided topic and objectives.

1) Given this broad topic, I am confused as to how so few studies were actually included (e.g. ref 1).

a. I am surprised that only a single research center was included in this report, given many research centers study the broad topic of psychological interventions in obesity.

b. It is unclear why “mindfulness” is listed as a search strategy intervention, but only two therapies were identified. Many seemingly relevant studies on mindfulness in adults with overweight/obesity are present in a current systematic review (ref 2) which were not included.

c. Search date/s needs to be provided to encompass all databases searched, beyond the example provided.

d. Given the search strategy, it is unclear why no studies were included prior to 2007.

e. Please better describe the screening process between 31,177 and 55 eligible studies. The search strategy does not align with other common reports in the field and the large, unexplained drop between 31,177 studies screened by an individual and the 55 eligible is concerning towards the validity of this search strategy.

f. Please provide the references that were excluded per:

i. Wrong intervention (n = 6)

ii. Wrong designation (n = 1)

iii. Wrong comparator (n = 3)

iv. Protocol only (n = 8)

2) Another critical, overarching concern is that weight loss and maintenance interventions, delivered particularly by registered dietitians, inherently incorporate standard of practice approaches, such as cognitive behavioral therapy (CBT) and motivational interviewing (MI). However, in the published reports of many dietitian-directed interventions, the explicit terms of CBT/MI may not be included in the methodology of the manuscripts; they may be contained within on-site study protocols or simply considered standard of practice and left out of text. This concept is present in Shaw 2005 – the article the authors self-reported to base this search strategy, whereby nutrition and/or physical activity interventions may employ psychological therapy interventions (without explicitly mentioning them) (ref 3).

There are notable studies that appear to fit this broad search, but are not present. These include, but are not limited to the landmark DASH diet trials, LOOK Ahead, Diabetes Prevention Program, PROPEL (delivered at primary care clinics), STAMPEDE (in combination with metabolic & bariatric surgery), perhaps even mobile health platforms such as VirtaHealth and WW. These interventions would theoretically fit under the broad category of the authors’ self-defined “Psychological therapy versus no intervention” or “usual care” as they consist of dietitian-led lifestyle interventions (ie., CBT/MI utilized), classically compared to a control group consisting of providing handouts of national dietary guidelines and exercise recommendations.

Towards these points:

3) Could the authors please comment on this overarching issue of psychological therapy being delivered as standard of practice and therefore not present in the text of their search?

a. With the understanding that it is not feasible to redo this search & review, I would be interested to hear the authors' opinion on why the notable studies that were mentioned above, many of which inherently include psychological therapy (CBT/MI) in the lifestyle intervention, were not present in this search and why there is such a large disconnect between the 31,000+ that were individually screened and 55 included.

REFERENCES

1. Morgan-Bathke M, Baxter SD, Halliday TM, Lynch A, Malik N, Raynor HA, Garay JL, Rozga M. Weight Management Interventions Provided by a Dietitian for Adults with Overweight or Obesity: An Evidence Analysis Center Systematic Review and Meta-Analysis. J Acad Nutr Diet. 2022. Epub 2022/07/06. doi: 10.1016/j.jand.2022.03.014. PubMed PMID: 35788061.

2. Grider HS, Douglas SM, Raynor HA. The Influence of Mindful Eating and/or Intuitive Eating Approaches on Dietary Intake: A Systematic Review. J Acad Nutr Diet. 2021;121(4):709-27.e1. Epub 20201203. doi: 10.1016/j.jand.2020.10.019. PubMed PMID: 33279464.

3. Tewksbury C, Nwankwo R, Peterson J. Academy of Nutrition and Dietetics: Revised 2022 Standards of Practice and Standards of Professional Performance for Registered Dietitian Nutritionists (Competent, Proficient, and Expert) in Adult Weight Management. Journal of the Academy of Nutrition and Dietetics. 2022;122(10):1940-54.e45. doi: 10.1016/j.jand.2022.06.008.

VERSION 1 - AUTHOR RESPONSE

**Reviewer comments**	**Responses**
**Reviewer: 1**<br>**Ms. Chiara De Poli, London School of Economics and Political Science**	
Abstract<br>Objectives: "psychological interventions and usual care" – this could be clarified as "psychological interventions vs usual care"?	We have revised as suggested to state psychological interventions versus usual care.
Abstract<br>Results – "thirteen psychological interventions for weight reduction in adults with overweight and obesity were identified" – should this read as "adults with overweight OR obesity"?	We have revised as suggested "adults with overweight or obesity".
Intro<br>Globally, the prevalence of obesity has risen to approximately 650 million (adults ≥18 years of age) - Could this sentence being reworded as "Globally,<br>the prevalence of obesity among the adult population (ie, individuals aged 18 and above) has risen to approximately 650 million" – or something to this effect	We have revised the sentence as suggested to improve clarity.

Intro<br>Can you also provide a definition for overweight and obesity	We have provided an additional sentence defining overweight and obesity.
Intro<br>- Overweight and obesity have a multifaceted aetiology, with factors such as genetics, biological influences, social components, medical conditions, stress, lifestyle factors, economic aspects, and environmental factors contributing to prevalence. – can these factors be represented in a more organic way and using some criteria, e.g. bio-medical vs socio-cultural, or individual- vs environmental-factors?	We have re-worded the sentence to categorise factors into individual and environmental (with examples).
Intro<br>"Over the past two decades, several interventions have been developed to tackle overweight and obesity which have largely focused on lifestyle modifications 7," – there has been a growing emphasis also on population based interventions to tackle overweight and obesity, such as various forms of sugar taxes, regulation of advertising, reform of licensing of food outlets etc. I think it is important to mention these alongside individual based interventions targeting lifestyle change.	We have provided additional text to highlight that there has been some policy-based approaches such as sugar taxes, regulation of advertising, reform of licensing of food outlets.
Intro<br>"While increasing energy expenditure and reducing dietary intake are critical for weight loss (in most cases), they often ignore the complex psychological,<br>emotional, and behavioural factors that underpin overweight and obesity" – I would argue that also broader environmental factors can contribute to<br>overweight and obesity – eg lack of safe parks and public areas for physical activity, lack of public transport that could be used as an alternative to driving,<br>exposure to advertising promoting unhealthy food, affordability of healthy food options, etc etc.	We have revised the sentence to include environmental factors alongside psychological, emotional, and behavioural factors.
Intro<br>p.3 L.46 "the psychological factors that contribute to overweight and obesity" – can you spell out these psychological factors. And what is the causal chain between these and overweight or obesity?	We have provided revised this paragraph to include your suggestions.
Intro<br>p.3 l.50 – I would avoid "etc	We have removed the word 'etc'.
Intro	We have revised the sentence to include a brief definition of weight reduction and weight

p.3 l.55 – what is the difference between weight reduction and weight management	management. This added text highlights the difference.
Intro<br>p.3 l.55-56 “In recent years, research in the field of weight reduction and weight management has rapidly evolved with a particular focus on psychological interventions” – can we explain why psychological interventions have been implemented more and more	We have added a sentence which explains why psychological interventions have been implemented more frequently in the past few years.
Intro<br>l.38 and 55, the two pars start in the same way “In recent years” – can you avoid the repetition?	We have replaced ‘in recent years’ with ‘Within the last two decades’ to avoid repetition.
Intro<br>p.3 l.58-59 “to assess the effectiveness of psychological interventions for weight reduction in adults with weight concerns” – what is meant by weight concern? Is the focus only weight reduction or also weight management mentioned above?	We have replaced the ‘weight concerns’ with ‘adults with overweight and obesity’. The focus of this review is on weight reduction.
Intro<br>p.4 “However, these reviews are limited by population (including only adults with specific medical conditions), type of interventions (including only one type of psychological therapy intervention), or being substantially outdated” – can you spell out the limitations of the reviews mentioned here and provide references for each. Eg what is meant by being substantially outdated? Why you think it is problematic that a review focused only on one type of psychological therapy intervention? What populations have been covered by previous reviews?	We have added details to each example i.e. specific medical conditions related to eating disorders, one type of psychological intervention being CBT and substantially outdated being a review by Shaw et al published in 2005 which is 19 years old.<br>We believe that by excluding certain intervention types, a systematic review may provide bias/ skewed conclusions and clinical recommendations not fully representative of the totality of evidence (which could be misinterpreted by a clinical reader).
Aim<br>- What is the difference between weight reduction and sustained weight reduction? Can you define both?	We have defined both in the ‘Types of interventions’ section of the methods.
Objectives<br>Objectives 1-3 are clear and consistent with the aim of the review (provided a definition of weight reduction and sustained weight reduction and of “adults”), I am not clear why among the objectives of the review three focus on usual care for weight reduction and sustained weight reduction (see objectives 4-6).<br>Can you please clarify/justify, since the scoping review does not map the results of the trials?	Thank you for the comment. We included usual care within our objectives because we intend to follow the scoping review with a full systematic review that will determine the effectiveness of psychological therapies for weight reduction. Generating a comprehensive map of the components of usual care will help us to define our inclusion criteria which we identified as a gap in knowledge within existing literature.
Search strategy	We adopted the approach to search from inception because we wanted to ensure that no

"We searched the above databases and trials registries from their inception to April 2023 (present)" – what was the added value of searching databases from inception? Were the definitions of overweight and obesity the same over this period? What was the state of the art of psychological interventions in the 50s compared to 2010s?	relevant studies were missed. This also was thought to provide us an opportunity to track the growth of evidence over time.<br>In addition, the "oldest" study included in this review was published in 2005 which likely reflects the recent focus on psychological factors. Therefore, given the World health Organisation descriptions of overweight and obesity in the early 2000's which focused on BMI, definitions then are not be different to the present day.
Can you justify why you have decided to include only Randomised controlled trials (RCTs) or non-randomised comparative intervention studies?	We only included Randomised controlled trials (RCTs) and non-randomised comparative intervention studies because the project was limited by funding and resource.<br><br>We acknowledge this as a limitation and have added additional sentences in the strengths and limitations section of the paper.
Can you specify that you consider for inclusion only interventions delivered by a professional – so it is clear that you are excluding those where a professional is not involved (eg interventions delivered via an app)	We have specified in the types of intervention section that we only included studies of psychological therapies that were delivered by a healthcare professional
Types of interventions<br>"Individual and group therapies were included" – Can this be reworded as "Interventions delivered one-to-one or in group setting were included"	We have reworded as suggested.
How did you deal with trials with a multi-pronged approach, e.g trials that combined a lifestyle intervention (eg providing advice on healthy diet, the importance of physical activity) and/or medications and/or a psychological intervention and/or multiple/integrated psychological interventions?	We have clarified in the methods that we only included studies with single component psychological therapy interventions, excluding those with multiple intervention components (e.g., psychological therapy combined with physical activity). We acknowledged this as a limitation and have made additions to the strengths and limitations section to reflect this.
For the comparison how was no intervention defined? No psychological intervention? No intervention of any type?	We have clarified in the type of intervention section that no intervention was no intervention of any type.
Outcomes<br>No psychological outcomes are listed here, which seems surprising (eg measures of self-efficacy, etc). can you please justify.	We have revised the section to state that we collected all available outcomes from the eligible studies which included psychological outcomes such as stress, anxiety, depression, health behaviour, body esteem, psychological function, psychological health, and self-efficacy.
- Table 1 though list secondary outcomes which are also psychological in nature. Can you please clarify in this section of the paper what outcomes intended to report?	In the protocol we listed some outcomes we anticipated to be included but in the full text we decided to report all outcomes outlined in the included studies. See table 1 and the mapping in the results section.

Data synthesis and analysis<br>- Can you provide details about Synthesis WIthout Meta-analysis in systematic reviews (SWiM)? Why did you think it was relevant for a scoping review?	We have removed the statement regarding SWiM as we did not rigorously follow its reporting items. Instead, we used it as a guideline but did not feel all items were relevant to this scoping review (i.e., items 5 and 6 regarding heterogeneity and effects).
Did you notice any differences in the definition of overweight and obesity used in each study/over time/in different countries?	We only included studies which used BMI as its criterion for overweight and obesity but did notice that studies differed in how this was determined i.e., height/weight measured by clinician, self-reported and self-measured.
Gaps within the evidence - You observe that “there is a notable absence of studies exploring therapy approaches such as counselling (integrative, eclectic), guided self-help, interpersonal therapy, cognitive analytic therapy, rational emotive behavioural therapy, exposure therapy, humanistic therapies, existential therapy, artistic therapies, music therapy, psychodrama, and systemic therapy” – did your search strings aim to identify such approaches/were your search strings broad enough to allow you identify studies using such approaches?	Thank you for the comment. We do believe our search strategy was comprehensive as it included 23 key word searches of different therapies including counselling, music therapy, humanistic therapy, rational emotive therapy behavioural therapy etc. We also included several MeSH terms which we believe would have captured most psychological therapies. Please see our MEDLINE search strategy in the supplementary material.
Discussion<br>I am not entirely sure about the aim of the comparison between psychological interventions vs usual care, since the results of the comparison are presented in a descriptive way (eg “Individuals receiving usual care received fewer appointments from their primary healthcare provider compared to those receiving psychological therapy interventions”), but no information is provided about the outcomes of usual care vs psychological interventions. Would not it be interesting to know what outcomes are achieved by each intervention arm or the difference between each intervention arm? If you think this is beyond the aim of a scoping review, why did you focus the review also on the comparators?	The scoping review is intended as a precursor to a systematic review to establish the effectiveness of psychological interventions for overweight or obesity. A comparison of effectiveness is outside the remit of a scoping review. We included usual care in the objectives of this review because generating a comprehensive map of the components of usual care will help us to define our inclusion criteria for the systematic review. We identified this as a gap in knowledge within existing literature that needed to be explored prior to conducting an SR in this area of research.
Discussion<br>The Discussion repeats some content included in the Gaps within the evidence – can this be streamlined? -	We have made an attempt to streamline the discussion section by removing some of the repetition relating to gaps in evidence.
Besides the clinical relevance of the interventions, do you want to comment on the feasibility of delivering **at scale** the interventions that you reviewed? Using the English context as an example, would the health	We have made additions to the third paragraph in the discussion to comment on feasibility and resources associated with delivering psychological therapy interventions.<br>We have stated that to determine whether these interventions could be delivered at scale

care system have capacity and resources for delivering these interventions at scale?	within the NHS, an economic evaluation would need to take place - depending on data available, we will consider conducting an economic effectiveness analysis alongside the clinical effectiveness analysis in our planned future systematic review.
Strengths and limitations - I think it should be explicitly acknowledged that the scope of the review excluded studies not designed as an RCT or a non-randomised intervention study. Studies such as those using qualitative research methods that aim to explore e.g. the feasibility of delivering psychological interventions in an overweight/obese population or the acceptability of such interventions among overweight/obese individuals and across individuals from different sociodemographic backgrounds (among whom maybe obesity is highly stigmatised) or studies that explain the take up of psychological interventions in an overweight/obese population (eg why people drop out from such interventions) were not included, despite the relevance of the evidence they can provide. Notably, all these aspects affect the effectiveness, uptake, etc of interventions tested out in RCTs	Thank you for the comment. We acknowledge this and have included additional sentences in the strengths and limitations section highlighting this as limitation.
Psychological interventions were predominantly delivered in multiple face-toface settings by healthcare professionals – can this be reworded as "Psychological interventions were predominantly delivered in multiple face-toface encounters by healthcare professionals based in different settings" – or something to this effect	We have revised this sentence as suggested.
**Reviewer: 2**<br>**Dr. Jonathan Hazlehurst, University of Birmingham**	
The abstract in its current form is not adequate and does not appropriately confirm with the PRISMA-ScR checklist. It needs to be re written. The authors may find this scoping review published in BMJ Open a useful reference for re writing the abstract From screen time to the digital level of analysis: a scoping review of measures for digital media use in children and adolescents | BMJ Open In particular in the results of the abstract it was not clear whether the authors found 13 different psychological interventions of 13 in total.	We have revised the abstract in accordance with the PRISMA-ScR checklist headings.<br><br>We have added clarity to the results section of the abstract by stating that a total of 31 studies met the inclusion criteria and that 13 unique psychological interventions were identified.
The introduction whilst otherwise well written is lacking a section on weight loss medications and bariatric surgery to better set the scene for the importance of psychological treatment.	Thank you for the comment. We have added an additional sentence in the introduction to acknowledge these as other weight loss interventions. That's said, whilst weight loss

	medications and bariatric surgery are interventions that play a part in some individuals weight loss journey's, we do not feel like these form part of the narrative of this paper. These interventions do not appear in the results, discussion, or conclusion and so we do not feel a more substantial mention of these is needed.
The search terms used, whilst appropriate for the bulk of the work, could not possibly hope to address outcome 4 and 5 given the search does not identify all "usual care" only studies incorporating a psychological treatment. This is a serious concern, especially given the published protocol paper in the same journal. If I am wrong and have missed something it should be addressed.	We acknowledge that because of our inclusion criteria we could not hope to generate a comprehensive overview of 'usual care' and have amended some of the verbiage used in the review to reflect this (i.e., removing comprehensive from the objective). We have provided an additional sentence in the methods that we will be looking at the usual care used in the papers extracted from the study selection process.<br>We have also acknowledged this with a further sentence in the 'limitations and strengths' section of the review.
In the results I see that 2 full text were excluded for "wrong comparator" yet there is not information given in inclusion or exclusion section about the comparator required. This needs to be justified.	We have clarified in the methods that studies were excluded if they included a comparison with other active lifestyle interventions. The exclusions relate to studies which the comparison was an active lifestyle intervention (e.g., physical activity sessions).
The PRIMSA flow chart includes* and ** with no legend for its interpretation	We have removed these.
Can the authors confirm that in Table 1 where an intervention is not specifically labelled as group it should be assumed to be individual	Thank you for highlighting this. We have added an asterisk and legend stating: delivered as an individual intervention unless specifically labelled as group delivery.
**Reviewer: 3**<br>**Dr. Jacob Mey, Pennington Biomedical Research Center**	
I read with interest the scoping review by Hamer et al on Psychological interventions for weight and maintenance in adults. I was particularly appreciative of the inclusion of a public advisor and the wide-spread use of person-first language (Notably, the article does still needs additional editing/double checking for person-first language, as instances of inappropriate use still remain e.g., Page 17 of 30, line 58 "who are … obese" should be "who have … obesity").	Thank you for highlighting this to us. We have checked the article and revised any instance whereby inappropriate language was used, and previously missed by our research team and public advisor.
1) Given this broad topic, I am confused as to how so few studies were actually included (e.g. ref 1).<br>a. I am surprised that only a single research center was included in this report, given many	The eligibility criteria narrowed the inclusion which was a limitation of this review. We have added further sentences to acknowledged some of these limitations in the 'strengths and limitations' section.

research centers study the broad topic of psychological interventions in obesity.	
b. It is unclear why "mindfulness" is listed as a search strategy intervention, but only two therapies were identified. Many seemingly relevant studies on mindfulness in adults with overweight/obesity are present in a current systematic review (ref 2) which were not included.	This may be due to our eligibility criteria e.g., exclusion of studies if they included a comparison with other active lifestyle interventions and exclusion of multi-component interventions. These have been clarified in the methods and acknowledged in the 'strengths and limitations' section.
c. Search date/s needs to be provided to encompass all databases searched, beyond the example provided.	Searches were conducted 19th April 2023 within all databases from inception of database to April 2023. We have clarified the date of searches in the methods section.
d. Given the search strategy, it is unclear why no studies were included prior to 2007.	This is due to our eligibility criteria, particularly the specific criteria surrounding population and intervention.
e. Please better describe the screening process between 31,177 and 55 eligible studies. The search strategy does not align with other common reports in the field and the large, unexplained drop between 31,177 studies screened by an individual and the 55 eligible is concerning towards the validity of this search strategy.	We have added additional detail in the methods and results to describe our screening and better highlight the process.<br><br>We now feel that the description of our screening process and results section exceeds the level of detail given in other scoping reviews published within the BMJ Open.<br>Please see Blanco D, Altman D, Moher D, et al. Scoping review on interventions to improve adherence to reporting guidelines in health researchBMJ Open 2019;9:e026589. Doi: 10.1136/bmjopen-2018-026589<br>And Jeyaraman MM, Copstein L, Al-Yousif N, et al. Interventions and strategies involving primary healthcare professionals to manage emergency department overcrowding: a scoping reviewBMJ Open 2021;11:e048613. doi: 10.1136/bmjopen-2021-048613
f. Please provide the references that were excluded per:<br>i. Wrong intervention (n = 6)<br>ii. Wrong designation (n = 1)<br>iii. Wrong comparator (n = 3)<br>iv. Protocol only (n = 8)	We have included a supplementary material (3) which details the excluded study references and reasons.
Another critical, overarching concern is that weight loss and maintenance interventions, delivered particularly by registered dietitians, inherently incorporate standard of practice approaches, such as cognitive behavioral therapy (CBT) and motivational interviewing (MI). However, in the published reports of many dietitian-directed interventions, the explicit terms of CBT/MI may not be included in the	This is a wider concern of all evidence synthesis projects given that any literature review would rely upon a search strategy comprised of mesh terms and keywords. If the interventions did not specify a psychological therapy of any type within the methodology of the manuscript, then it would not be picked up during database searching. We have attempted to mitigate this

methodology of the manuscripts; they may be contained within on-site study protocols or simply considered standard of practice and left out of text. This concept is present in Shaw 2005 – the article the authors self-reported to base this search strategy, whereby nutrition and/or physical activity interventions may employ psychological therapy interventions (without explicitly mentioning them) (ref 3).	by including citation searching within our search strategy.
There are notable studies that appear to fit this broad search, but are not present. These include, but are not limited to the landmark DASH diet trials, LOOK Ahead, Diabetes Prevention Program, PROPEL (delivered at primary care clinics), STAMPEDE (in combination with metabolic & bariatric surgery), perhaps even mobile health platforms such as VirtaHealth and WW. These interventions would theoretically fit under the broad category of the authors' self-defined "Psychological therapy versus no intervention" or "usual care" as they consist of dietitian-led lifestyle interventions (ie., CBT/MI utilized), classically compared to a control group consisting of providing handouts of national dietary guidelines and exercise recommendations.	Thank you for the comment. We have screened the studies mentioned and based on our specific inclusion criteria none would be included. Although these are important studies, they have been excluded due to our specific PICO's. The reasons are as follows:<br>The Diabetes prevention program did not meet out inclusion because it included participants with a BMI of 22 to 24kg/$m^{2.}$<br>The DASH trial was not included because its intervention was a diet assigned to participants. The psychological component described in the intervention did not meet the criteria of inclusion of our review (because it was not defined).<br>The PROPEL study includes an education and training intervention and not a psychological therapy intervention.<br>The STAMPEDE trail included an active comparison including components of weight management (physical activity) and drug therapy and so did not meet the inclusion criteria.<br>The LOOK Ahead trial included an intervention with components of physical activity and the comparator included diabetes support which we did not consider 'usual care' for all adults with overweight or obesity.<br>VirtaHealth was excluded because it was delivered online and did not include participants meeting with a professional to undertake a psychological therapy intervention. We were unsure what WW referred too so we could not screen the paper.<br>That said, we acknowledge that the review does have some limitations regarding the eligibility criteria, and we have added these in the 'strengths and limitations' section.
Could the authors please comment on this overarching issue of psychological therapy being delivered as standard of practice and therefore not present in the text of their search?	We believe this is a wider limitation of all evidence synthesis projects given that any literature review would rely upon a search strategy comprised of mesh terms and

	keywords. If the interventions did not specify a psychological therapy of any type within the methodology of the manuscript (or background, results/ conclusion), then it would not be picked up during database searching. Only a comprehensive full text screening of all relevant articles would mitigate this concern. Funding limitations associated with this project meant that we could not perform a full text screen of all 31,000+ articles.
a. With the understanding that it is not feasible to redo this search & review, I would be interested to hear the authors' opinion on why the notable studies that were mentioned above, many of which inherently include psychological therapy (CBT/MI) in the lifestyle intervention, were not present in this search and why there is such a large disconnect between the 31,000+ that were individually screened and 55 included.	The review has a specific inclusion criterion which excluded the studies above.<br>We have attempted to describe the study selection process in greater depth in the methods section to address the disconnect between the 31,000+ and the 31 included studies.<br>We acknowledged that the review does have some limitations (as do all reviews) and we have added these in the 'strengths and limitations' section.

VERSION 2 - REVIEW

Reviewer 1

Name De Poli, Chiara

Affiliation London School of Economics and Political Science, Management

Date 29-Apr-2024

COI no competing interests

Thanks for the opportunity to read a revised version of the article "Psychological interventions for weight reduction and sustained weight reduction in adults with overweight and obesity: a scoping review". The authors have satisfactorily addressed the comments raised. I offer a few minor editorial comments for the authors' consideration.

Introduction

- *"Over the past two decades, several interventions have been developed to tackle overweight and obesity which have largely focused on individual-level lifestyle modifications (e.g., increasing physical activity) as well as population level policy-based approaches (e.g., sugar taxes, regulation of advertising, reform of licensing of food outlets) (7,8). Although most interventions for weight reduction have focused on lifestyle modifications, some have included surgery (bariatric surgery) and pharmacological medication (6,7,8)"* – Could this be reworded as "Over the past two decades, although most interventions for weight reduction have focused on individual-level lifestyle modifications (e.g., increasing physical activity), some have included surgery (bariatric surgery) and pharmacological medication (6,7,8). These have been accompanied by policy-based approaches (e.g., sugar taxes, regulation of advertising, reform of licensing of food outlets) (7,8) aiming to address obesity at the population level"

- Par "*While increasing energy expenditure and reducing dietary intake (13) are critical for weight loss (in most cases), they often ignore the complex psychological, emotional, environmental, and behavioural factors that underpin overweight and obesity (.2,14-16). These factors can contribute to disordered eating, low selfesteem, depression, anxiety, and stress, which in turn can lead to further weight gain*" - It may be good to spell out why environmental factors contribute to low self esteem, depression, anxiety

Methods

- Study selection. How have the Authors handled articles labelled as undecided?

Outcomes

- "*As a consequence, there is little known about the potential efficacy of these psychological therapy interventions*" - This could be reworded "little is known about the potential effectiveness...". It seems appropriate to replace efficacy with effectiveness throughout the paper (see also in the discussion session, p20)

Discussion

- "*This review provides an up-to-date synthesis of existing therapy interventions used to promote weight reduction in adults with overweight and obesity. (20,23,67*)" – this sentence could be moved right at the start of the discussion, it could be the first sentence in the discussion section.
- *"This review highlighted several key evidence gaps within existing literature. This is consistent with findings of other reviews"* – could this be reworded as "This

review highlighted several key evidence gaps which are consistent with findings already available in the literature"?

Strengths and limitations

- "One of the notable strengths of this scoping review is the methodology employed. Given the objectives of this review, utilising a scoping review methodology allowed for a more expansive inclusion criteria, ensuring all relevant studies relating to psychological therapy interventions for adults with overweight and obesity could be included". – Can you please clarify compared to what the scoping review methodology allowed for *more* expansive inclusion criteria?

**Reviewer**	**3**
**Name**	**Mey, Jacob**
**Affiliation**	**Pennington Biomedical Research Center**
**Date**	**16-Apr-2024**
**COI**	**n/a**

I appreciate the authors' revisions and responses.

However, the primary issue remains to be adequately addressed.

Through their response, the authors have largely indicated that the critical issues raised by reviewers are inherent to their search methods (mentioned as a limitation in response to each of the 3 peer reviewers). As one of these reviewers, I agree that the search methods are perhaps THE critical issue and thus the final search may not adequately represent the literature.

Per the authors: "We now feel that the description of our screening process and results section exceeds the level of detail given in other scoping reviews" - I again agree. However, the problem remains that a highly detailed description of an inadequate search does not improve or address the issues of using an inadequate search in the first place.

It should be noted that this search includes 31,177 articles for screening, yet only 57 full texts reviewed and 31 final included studies. These search numbers are abnormal and not consistent with other scoping reviews, even specifically in BMJ Open.

Further, the authors provided 2 example comparative reviews in BMJ Open:

Example 1) 1399 screened and 435 full texts reviewed for 31 final included studies (Blanco 2019)

Example 2) 20,663 screened and 2291 full text reviews for 247 final included studies (Jeyaraman 2021).

Please note how the the Blanco study, that included a final number of studies similar to this report, screened nearly 30,000 less studies in their report. Please note also, how the Jeyaraman study, that screened a number of studies similar to this report (although Jeyaraman still screened ~10,000 less studies than this report), reviewed >2200 more full texts and included >200 more final studies compared to this report.

These profound discrepancies point to critical, potentially fatally flawed issues in the search criteria and methods. These concerns are large enough, that the superficial responses by the authors (stating in simplified language that these concerns are just inherent to the search) are inadequate to curtail them.

Of note, even in the authors' response to reviewers, they appear unclear on how their search functioned. For specific example, the authors state, "If the interventions did not specify a psychological therapy of any type within the methodology of the manuscript, then it would not be picked up during database searching." Yet, in their specific exclusion rationale for multiple studies, the authors state "no psychological [therapy]." Both of these statements cannot be true if the search functioned as the authors described (i.e., meaning the articles were 'picked up during database searching' despite not specifying a psychological therapy). This is a mere example of the issue that >30,000 articles underwent title & abstract screening for only 31 included studies. Something is amiss with the search or screener exclusion such that the final included articles are not likely representative of the literature for what the authors propose.

I urge the authors to provide an improved explanation for their abnormal search numbers and better justify their search strategy - as unfortunately, in the current state, I am not confident that this search and most critically, the conclusions drawn based upon conducting an effective search, are representative of the literature as its described.

VERSION 2 - AUTHOR RESPONSE

We wish to thank the reviewers for the comments and resubmit our manuscript titled "Psychological interventions for weight reduction and sustained weight reduction in adults with overweight and obesity: a scoping review" with amendments. We have now addressed all comments made the reviewers and responded in the attached document.

Reviewer comments and author responses - Bmjopen-2023-082973.R1 manuscript

Reviewer 1 comments	Author response
**Introduction**<br>"Over the past two decades, several interventions have been developed to tackle overweight and obesity which have largely focused on individual-	Thank you for the comment, we have re-worded as suggested.

level lifestyle modifications (e.g., increasing physical activity) as well as population level policy based approaches (e.g., sugar taxes, regulation of advertising, reform of licensing of food outlets) (7,8). Although most interventions for weight reduction have focused on lifestyle modifications, some have included surgery (bariatric surgery) and pharmacological medication (6,7,8)" – Could this be reworded as:<br>"Over the past two decades, although most interventions for weight reduction have focused on individual-level lifestyle modifications (e.g., increasing physical activity), some have included surgery (bariatric surgery) and pharmacological medication (6,7,8). These have been accompanied by policy-based approaches (e.g., sugar taxes, regulation of advertising, reform of licensing of food outlets) (7,8) aiming to address obesity at the population level"	
Par "While increasing energy expenditure and reducing dietary intake (13) are critical for weight loss (in most cases), they often ignore the complex psychological, emotional, environmental, and behavioural factors that underpin overweight and obesity (.2,14-16). These factors can contribute to disordered<br>eating, low self-esteem, depression, anxiety, and stress, which in turn can lead to further weight gain" - It may be good to spell out why environmental factors contribute to low self esteem, depression, anxiety	We have added an example to clarify at the end of the paragraph on page 4 'environmental factors such as access to safe and non-discriminatory recreational spaces, and access to healthy, affordable food may exacerbate psychological struggles'
**Methods**<br>Study selection. How have the Authors handled articles labelled as undecided?	We have clarified that a second reviewer was asked to screen studies which were initially categorised as undecided.
**Outcomes**<br>"As a consequence, there is little known about the potential efficacy of these psychological therapy interventions" - This could be reworded "little is known about the potential effectiveness…". It seems appropriate to replace efficacy<br>with effectiveness throughout the paper (see also in the discussion session, p20)	We have revised the wording as suggested, replacing efficacy<br>with effectiveness in the outcomes and discussion.
**Discussion**<br>"This review provides an up-to-date synthesis of existing therapy interventions used to promote weight reduction in adults with overweight and obesity. (20,23,67)" – this sentence could be moved right at the start of the discussion, it could be the first sentence in the discussion section.	We have moved this sentence to the beginning of the discussion as suggested.
- "This review highlighted several key evidence gaps within existing literature. This is consistent with findings of other reviews" – could this be reworded as "This review highlighted several key	We have re-worded as suggested.

evidence gaps which are consistent with findings already available in the literature"?	
Strengths and limitations<br>- "One of the notable strengths of this scoping review is the methodology employed. Given the objectives of this review, utilising a scoping review methodology allowed for a more expansive inclusion criteria, ensuring all relevant studies relating to psychological therapy interventions for adults with overweight and obesity could be included". – Can you please clarify compared to what the scoping review methodology allowed for *more* expansive inclusion criteria?	We have clarified that the scoping review methodology allowed for a more expansive inclusion criteria compared with a systematic review methodology. We have provided an additional sentence in the strengths and limitations section to clarify this.
**Reviewer 3 comments**	**Author comments**
I appreciate the authors' revisions and responses.<br><br>However, the primary issue remains to be adequately addressed.<br><br>Through their response, the authors have largely indicated that the critical issues raised by reviewers are inherent to their search methods (mentioned as a limitation in response to each of the 3 peer reviewers). As one of these reviewers, I agree that the search methods are perhaps THE critical issue and thus the final search may not adequately represent the literature.<br><br>Per the authors: "We now feel that the description of our screening process and results section exceeds the level of detail given in other scoping reviews" - I again agree. However, the problem remains that a highly detailed description of an inadequate search does not improve or address the issues of using an inadequate search in the first place.<br><br>It should be noted that this search includes 31,177 articles for screening, yet only 57 full texts reviewed and 31 final included studies. These search numbers are abnormal and not consistent with other scoping reviews, even specifically in BMJ Open.<br><br>Further, the authors provided 2 example comparative reviews in BMJ Open:<br>Example 1) 1399 screened and 435 full texts reviewed for 31 final included studies (Blanco 2019)<br>Example 2) 20,663 screened and 2291 full text reviews for 247 final included studies (Jeyaraman 2021).	Thank you for your feedback on our manuscript. We appreciate your comments regarding the search strategy and screening process, and we would like to provide further clarification on these points to address your concerns.<br><br>We understand your concern regarding the screening from 31177 hits to 31 selected, as this does not follow the "usual 10% left at each stage" norm. We believe the final selection of 31 studies does represent the available evidence specific to psychological interventions for the purpose and population detailed in this review, without the inclusion of other components (e.g., physical activity or diet). That said, we have acknowledged this concern as a limitation in the discussion.<br><br>We feel it is important to highlight that the vast majority of the screened studies were excluded because they included some form of lifestyle component in either the intervention or comparator groups (e.g., dietary changes or physical activity), which was outside the scope of this review. In general, weight management literature is heavily dominated by studies combining psychological interventions with a range of lifestyle components. The small number of included studies can be attributed to the specific inclusion and exclusion criteria we adopted (acknowledged in the limitations section of the review). This criteria was extensive:<br>• We excluded studies with multiple intervention components (e.g., psychological therapy combined with physical activity or diet)

Please note how the the Blanco study, that included a final number of studies similar to this report, screened nearly 30,000 less studies in their report. Please note also, how the Jeyaraman study, that screened a number of studies similar to this report (although Jeyaraman still screened ~10,000 less studies than this report), reviewed >2200 more full texts and included >200 more final studies compared to this report.<br><br>These profound discrepancies point to critical, potentially fatally flawed issues in the search criteria and methods. These concerns are large enough, that the superficial responses by the authors (stating in simplified language that these concerns are just inherent to the search) are inadequate to curtail them.<br><br>Of note, even in the authors' response to reviewers, they appear unclear on how their search functioned. For specific example, the authors state, "If the interventions did not specify a psychological therapy of any type within the methodology of the manuscript, then it would not be picked up during database searching." Yet, in their specific exclusion rationale for multiple studies, the authors state "no psychological [therapy]." Both of these statements cannot be true if the search functioned as the authors described (i.e., meaning the articles were 'picked up during database searching' despite not specifying a psychological therapy). This is a mere example of the issue that >30,000 articles underwent title & abstract screening for only 31 included studies. Something is amiss with the search or screener exclusion such that the final included articles are not likely representative of the literature for what the authors propose.<br><br>I urge the authors to provide an improved explanation for their abnormal search numbers and better justify their search strategy - as unfortunately, in the current state, I am not confident that this search and most critically, the conclusions drawn based upon conducting an effective search, are representative of the literature as its described.	• We excluded studies with comparisons with other active lifestyle interventions (physical activity/ diet, not defined as usual care).<br>• We excluded studies that we could not retrieve a full text.<br>• We excluded non-peer reviewed articles.<br>• We excluded studies not available in English.<br>• We included studies in which the psychological therapy was delivered by a healthcare professional.<br>• We included studies whereby the intervention was designed to reduce weight or increase health promoting behaviour for the purpose of weight reduction and sustained weight reduction.<br>• We included randomised controlled trials (RCTs) and non-randomised comparative intervention<br>• We included studies whereby a psychological therapy intervention were defined as those interventions that involved meeting with a therapist.<br>• We included studies if the participants were ≥18 years and were classified as overweight or obese (identified by BMI ≥ 25kg/m^2)<br><br>With regard to our search, we feel our search followed the standard guidance for conducting searches for reviews (Cochrane Handbook etc.) and was conducted by an experienced information specialist. We searched for terms related to Population and Intervention and used a recognised search filter to limit the search by study design (CADTH - RCTs and non-randomised controlled trials). This is in line with published guidance on conducting searches for reviews:<br>• https://www.eunethta.eu/wp-content/uploads/2020/01/EUnetHTA_Guideline_Information_Retrieval_v2-0.pdf (see section 3.1.3)<br>• https://sites.google.com/york.ac.uk/sureinfo/home/search-strategy-development<br>• https://training.cochrane.org/handbook/current/chapter-04-technical-supplement-searching-and-selecting-studies

	We also based our search terms on a search conducted in a previously published Cochrane Review (conducted 20 years ago and the sizes of the databases have increased vastly since then: https://www.nlm.nih.gov/bsd/medline_cit_counts_yr_pub.html).<br><br>With regard to the database search, it is likely that the high number of hits retrieved is down to the population and intervention concepts being very broad (e.g., overweight, obesity and psychological interventions). As we were including any type of psychological intervention it was necessary to include subject headings and search terms for all possible types of psychological intervention – of which there are many. Obesity is also a very broad concept, and these are common terms which are not unique to the population, i.e., a lot of abstracts mention terms for obesity/ overweight without it being the main population of the study. Another important factor to consider is that some databases such as Embase tend to return a particularly high number of hits, but the subject indexing is less accurate (it is automated, and a larger number of subject terms are applied to each abstract reducing accuracy) and therefore it tends to return a higher number of irrelevant results when searching subject headings. In addition, it would have been extremely difficult to include terms for comparators in the search without seriously impacting on the return of relevant studies. Equally, we did not include search terms for outcomes because these are not usually included in review searches as these are not always explicitly described or adequately indexed in abstracts (see guidance above). In hindsight, we agree that we could have added additional search limits, such as excluding child and young people (age group limits) and refined our search terms, which would have reduced the initial number of articles identified and made screening more efficient. This process would have very likely made the search numbers similar to other reviews. We have acknowledged this as a limitation in the discussion.<br><br>We appreciate your observations regarding the potential confusion around our statement: "If the interventions did not specify a psychological therapy of any type within the methodology of the manuscript, then it would

	not be picked up during database searching." To clarify, our search strategy was built around key terms related to psychological therapies, so any study mentioning these terms would be retrieved. However, during full-text screening, we excluded studies whereby psychological therapies were not explicitly implemented as per our inclusion criteria (e.g., the abstract may have led us to believe the intervention was a psychological therapy intervention with a therapist or without additional components, but the full texts revealed that there was not the presence of a therapist or that other lifestyle components accompanied it). Therefore, while some articles may have appeared in the initial search and included during initial screening due to broad search terms, they were excluded during the more stringent full-text screening process. In summary, the database search would find articles that mentioned psychological therapy, but we subsequently excluded studies whereby the intervention did not meet our inclusion definition, where it was combined with other active lifestyle interventions and/ or the comparator was an active lifestyle intervention (among other exclusion criteria).
